# Association between Maternal Lead Exposure from Seafood Consumption and Neurodevelopment: A Systematic Review

**DOI:** 10.1016/j.advnut.2025.100380

**Published:** 2025-01-21

**Authors:** Arin A Balalian, Maureen K Spill, Rachel C Thoerig, Rupal Trivedi, Sanjoy Saha, Margaret J Foster, Amanda J MacFarlane

**Affiliations:** 1 Texas A&M Agriculture, Food and Nutrition Evidence Center, Fort Worth, TX, United States; 2 Center for Systematic Reviews and Research Syntheses, University Libraries, Texas A&M University, College Station, TX, United States; 3 Department of Nutrition, Texas A&M University, College Station, TX, United States

**Keywords:** lead, pregnancy and lactation, seafood, fish, neurodevelopment, cognition, Pb

## Abstract

Despite the essential nutrients, maternal seafood consumption during pregnancy or lactation (PL) is also a potential source of toxins, including lead (Pb). The association between exposure to Pb from seafood during PL on children’s neurodevelopment is uncertain. This systematic review assessed the association of exposure to Pb from seafood during PL on children’s neurodevelopment. Embase, PubMed, CENTRAL, and PsycINFO were searched for English-language peer-reviewed articles. Two independent reviewers screened at title, abstract, and full-text levels. Experimental and observational studies comparing different levels of exposure to seafood and Pb were eligible if: *1*) the exposed population included healthy pregnant or lactating individuals from high or very high human development index countries; *2*) assessed neurodevelopment in children ≤18 y old; and *3*) measured maternal seafood intake, Pb exposure and analyzed their relationship with each other and/or their association with child neurodevelopment. The Cochrane risk of bias in nonrandomized studies - of exposure (ROBINS-E) and Grading of Recommendations Assessment, Development, and Evaluation (GRADE) tools were used to assess the risk of bias and certainty of evidence. Four included articles from 3 prospective cohort studies in Asia examined cognition, motor development, and behavior in children 12–60 mo. Only 1 study reported an analysis between seafood intake and Pb concentrations during PL, which showed a weak, nonsignificant association. Pb concentrations were not associated with child cognitive development or behavioral problems, but a weak, negative association with child motor development was reported. The certainty of the evidence was very low due to the few included studies with some or a high risk of bias. Higher seafood intake in this evidence favored positive developmental outcomes from 1 prospective cohort study, though significance varied. Overall, evidence was not available to address a direct association between Pb exposure from seafood intake during PL and child neurodevelopment. Several other limitations resulted in a very low certainty of overall evidence.

This systematic review was registered at PROSPERO as CRD42023494884.

**Statements of significance**

This systematic review assessed the association between exposure to lead (Pb) from seafood during pregnancy and child neurodevelopment. Based on 4 studies, there was no significant association between Pb concentrations during pregnancy and child cognitive and behavioral issues. There was a significant but weak association between Pb concentrations and motor development. The certainty of evidence is very low due to the few studies included and the risk of bias concerns.

## Introduction

The perinatal phase is a time when vital macro and micronutrients required for development are acquired directly from the mother during pregnancy or breastfeeding [1]. Seafood provides a significant source of essential nutrients such as iodine, vitamin B12, iron, vitamin D, zinc, manganese, and omega (ω)-3 and ω-6 fatty acids [1,2]. Some of these nutrients are particularly beneficial for the growth and development of children.

Beyond its nutritional benefits, seafood is a potential route of exposure to various environmental contaminants, including lead (Pb) [3,4]. Pb is a naturally occurring, toxic heavy metal [5,6]. Once it enters the body, Pb can pass the blood-brain barrier [7,8]. In the brain, even in small amounts, Pb impairs the storage and release of neurotransmitters and consequently disrupts crucial processes of neurodevelopment, such as proliferation, differentiation, and synapse formation of neurons [7,8]. Research in animal models suggests that Pb has a long half-life in the brain, adding to the concern of Pb ingestion. Although the association between prenatal exposure to Pb from any source and neurodevelopmental outcomes such as cognition, behavior, and risk of attention deficit hyperactivity disorder is well-established [[9], [10], [11], [12], [13], [14]], there is a paucity of information regarding exposure to Pb specifically from seafood sources and its association with child neurodevelopment.

In order to refine dietary guidelines, it is important to understand both the benefits and risks of consuming seafood, particularly during pregnancy and lactation (PL), on child development. This systematic review, part of a series of reviews, was conducted to inform the National Academies of Sciences, Engineering, and Medicine (NASEM) expert Committee on “The Role of Seafood Consumption in Child Growth and Development” [15]. Our recent scoping review identified peer-reviewed literature related to the exposure of various toxicants from seafood consumed during PL or during childhood and adolescence on child developmental and health outcomes [16]. Although there is no minimum number of studies required for a systematic review, for the purposes of this project and in consultation with the NASEM expert committee, “sufficient evidence” for a seafood toxicant-outcome pair was defined as 3 or more studies. Only a few toxicant-child outcome pairs were identified to have sufficient literature (≥3 studies) to be considered for systematic review, including Pb and neurodevelopment [16]. Thus, the purpose of this systematic review was to identify, assess, and synthesize the evidence on the relationship between Pb exposure from maternal seafood consumption during PL and the neurodevelopment of the child to inform dietary guidelines.

## Methods

### Protocol and search strategy

This systematic review protocol was registered in PROSPERO (CRD42023494884). After the protocol was registered, a clarification for inclusion and exclusion criteria was added, specifically that to be included, studies had to assess the associations between toxicant and seafood exposure and neurodevelopmental outcomes. Upon completion of the review, we followed the PRISMA reporting guidelines for transparency (Supplemental Table 1). An experienced systematic review librarian (MJF) conducted a search in Embase, PubMed, PsycINFO, and CENTRAL in October 2024 (Supplemental Table 2).

### Study selection

Two researchers independently screened the studies at the title, abstract, and full-text levels utilizing DistillerSR software [17] based on the inclusion and exclusion criteria. The inclusion and exclusion criteria were developed in consultation with experts from the NASEM committee and are summarized in Supplemental Table 3. Briefly, eligible studies had to be conducted in countries classified as high or very high on the human development index, as these are the criteria used for systematic reviews that inform the dietary guidelines for Americans. Studies were required to assess both seafood consumption and Pb exposure during pregnancy and/or lactation. Additionally, they needed to perform an analysis to determine if there was a relationship between Pb and seafood exposure as well as between each, Pb and seafood, on neurodevelopmental outcomes (cognition and motor development, behavioral issues, attention, and autism spectrum disorders). Alternatively, studies were considered eligible if they investigated the effects of both Pb and seafood on neurodevelopmental outcomes, even if the direct relationship between the 2 exposures was not explicitly reported (Supplemental Figure 1). Furthermore, studies were required to compare Pb exposure across a range of concentrations, including no exposure. For seafood intake, comparisons were based on different types, sources, amounts, frequencies, durations, preparation methods, or timings of consumption, including no seafood intake. Eligible study designs included prospective and retrospective cohort studies, case-cohort studies, case-control studies, before-and-after studies, quasi-experimental designs, and randomized controlled trials. Conflicts were resolved by the reviewers, or when necessary, a third reviewer was consulted. The reference lists of the included studies were also screened manually to include any relevant articles.

### Data extraction and risk of bias assessments

All data were extracted by an experienced, trained analyst using a standard extraction form. A second analyst reviewed all extracted data for accuracy and completeness. All studies underwent dual, independent risk of bias (RoB) assessments using ROBINS-E (a tool designed for assessing RoB in nonrandomized studies of exposure) [18]. To assess the RoB due to confounding several key confounders [i.e., child: sex, age, race/ethnicity; parental: *socioeconomic status*, smoking, education, alcohol consumption, weight, height, BMI (in kg/m^2^); infant feeding mode; nonseafood dietary exposure to n–3 PUFAs; family history of the outcome] were considered based on a literature review and in consultation with experts from the NASEM committee. For questions pertaining to exposure assessments, both exposures (seafood and Pb) were considered, and, to be conservative, the higher RoB rating was recorded. Conflicts were reviewed and resolved by a third reviewer, if necessary. All domains within ROBINS-E were assessed, and an overall RoB rating was assigned to each study.

### Data synthesis and analysis

A qualitative synthesis was conducted and reported by a specific outcome domain. The presence or absence of an association was determined based on the magnitude of the effect and the width of 95% confidence intervals (CI). If the magnitude of the effect was between –1.0 and 1.0 (close to null = 0) and the 95% CI included the null, we concluded that there was no association. If the magnitude of the effect was larger than –1.0 or 1.0, and the 95% CI included the null value, we determined that the association was not significant. We did not conduct a pooled analysis due to considerable clinical, methodological, and statistical heterogeneity [19]. A certainty of evidence rating was determined using the Grading of Recommendations Assessment, Development, and Evaluation (GRADE) approach [20]. Because of the small number of studies included in this systematic review, we did not perform a sensitivity analysis.

## Results

**FIGURE 1:**
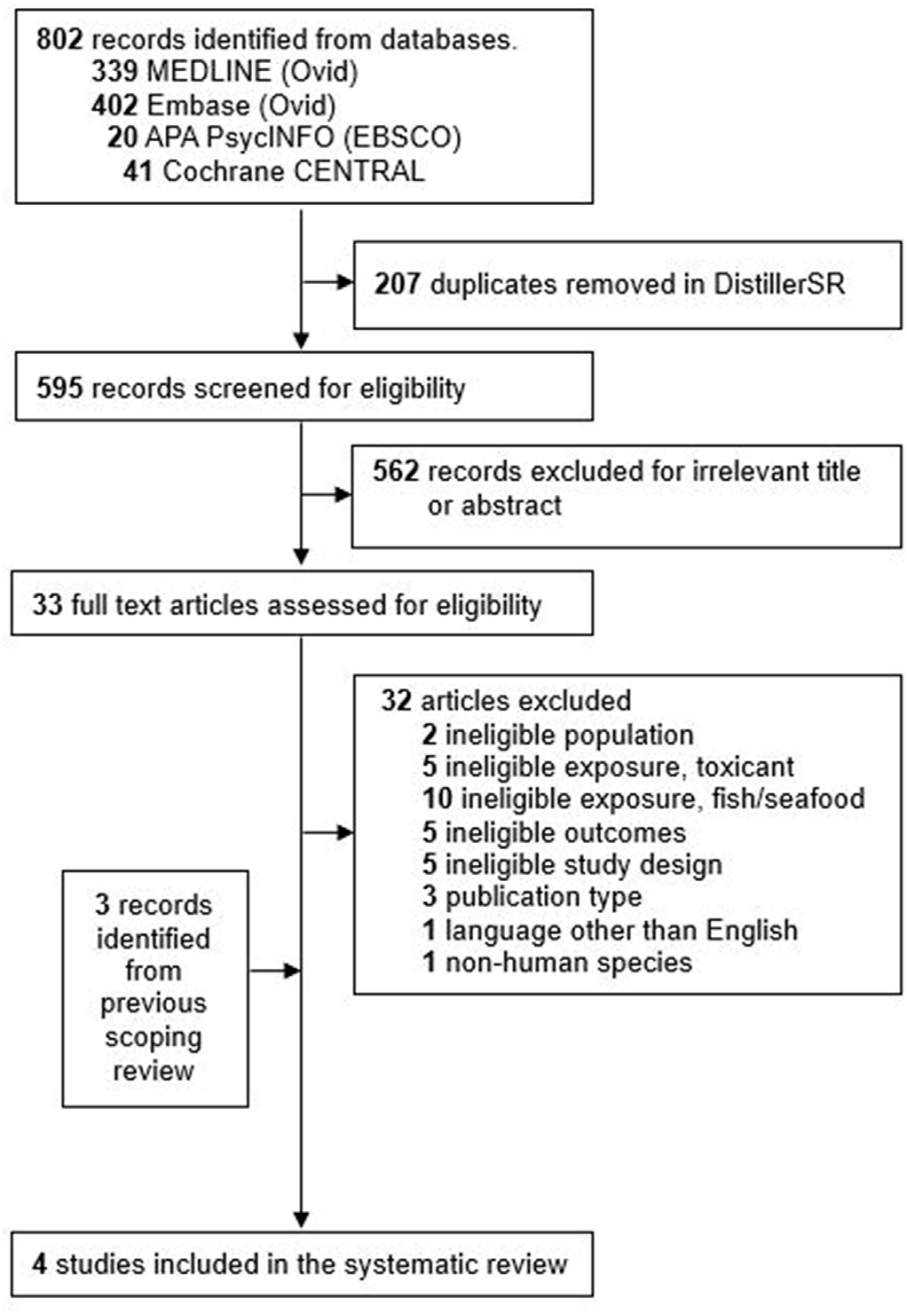
PRISMA flow diagram of screened and selected studies.

Overall, 802 articles were identified in the electronic database search. Four articles from 3 prospective cohort studies were included in the review (Figure 1). Two articles were based on the same cohort (Daxin County cohort) in China [21,22], 1 study was conducted in the Tohoku Study of Child Development cohort in Japan [23], and the last in mothers’ and children’s environmental health study in Korea [24]. All 4 articles provided an analysis of maternal seafood intake and neurodevelopmental outcomes as well as maternal Pb concentrations and neurodevelopmental outcomes; however, only 1 article assessed the relationship between maternal seafood intake and Pb concentrations, which indicated a weak and nonsignificant correlation (r < 0.1) [21].

### Seafood intake

The details about seafood intake and Pb exposure are summarized in Table 1 [21,22,25,26]. The included articles reported seafood intake using various units. Thus, we converted the seafood intake units to grams per week (g/wk) for interstudy comparisons (Table 1). Exposure to seafood was assessed using validated food frequency questionnaires (FFQs) in 3 articles from 2 cohort studies [21,22,25]. These articles assessed the intake and frequency of a wide variety of seafood in the third trimester [21,22] or after delivery [25]. Jeong et al. [26] (2017) did not use a validated FFQ to assess seafood intake; however, this article indicated that a detailed questionnaire was used to assess exposure and other factors. Only 1 article reported the type of seafood assessed as fish and shellfish [21]. Seafood intake varied across the studies. In the Korean study, seafood consumption was relatively low (66.5% consumed 0–75 g/wk of seafood) [26]. Seafood consumption was moderate in the Chinese cohort (46% consumed 0–340 g/wk of seafood) [21,22] and highest in the Japanese study (median 392 g/wk) [25].

**TABLE 1:** Characteristics of included studies.

Article identifier; name of the study/cohort; country	Pb exposure assessment mode, levels, and time	Time and method of seafood intake assessment	Seafood intake *n* (%)^1^	Outcome assessment: tool; child age; analytic *n*	Findings: Pb and neurodevelopment	Findings: seafood intake and neurodevelopment
Cognitive development						
Jeong, 2017 [26] Mothers and children’s environmental health study; Republic of Korea	Maternal blood (NR), late pregnancy	Late pregnancy, dietary questionnaire	≤20.5 g/wk: 148 (33.1%) 20.5–75.0 g/wk: 149 (33.4%) >75.0 g/wk: 149 (33.4%)	K-WPPSI; 60 mo; *n* = 553	Pb not associated with WPPSI scores, data NR	No association between average child IQ scores and maternal fish intake, by tertiles, mean (SD); 1. Verbal IQ First tertile: 103.4 (14.5) Second tertile: 102.0 (13.5) Third tertile: 103.6 (14.7) *P* = 0.543 2. Performance IQ First tertile: 101.8 (14.5) Second tertile: 102.5 (18.4) Third tertile: 102.3 (15.7) *P* = 0.932 3. Total IQ First tertile: 103.0 (16.2) Second tertile: 105.1 (15.1) Third tertile: 103.5 (15.2) *P* = 0.896
Rothenberg, 2016 [21] Daxin County cohort; China	Maternal blood median (min, max) [2.6 *μ*g/dL (0.96, 7.8)], peripartum	Third trimester, the validated food frequency questionnaire	0 g/wk: 169 (43%) 0–340 g/wk: 178 (46%) ≥340 g/wk: 44 (11%)	BSID-II: MDI; 12 mo; *n* = 270	Nonsignificant negative association between Pb measured in maternal blood and cognitive development, β (95% CI): −3.7 (−12, 4.8), *P* > 0.05	Significant positive association with cognitive development if seafood intake ≥ twice/wk, β (95% CI): 0/wk ref. 0 < ingestion < twice/wk: 1.7 (−0.86, 4.2) Ingestion ≥ twice/wk: 4.1 (0.04, 8.2)
Rothenberg, 2021 [22] Daxin County cohort; China				BSID-II: MDI; 12 mo; *n* = 264; 36 mo; *n* = 190	No association between Pb measured in maternal blood and cognitive development at 12 mo and 36 mo, β (95% CI): 0.11 (−2.0, 2.2), *P* > 0.05	Significant positive association with cognitive development if seafood intake ≥2 svg/wk, β (95% CI): 0 svg/wk: ref. 0< svg/wk <2: 1.7 (−0.34, 3.7), *P* > 0.05 ≥2 svg/wk: 4.7 (1.4, 8.0), *P* < 0.01
Motor development						
Rothenberg, 2016 [21] Daxin County cohort; China	Maternal blood median (min, max) [2.6 *μ*g/dL (0.96, 7.80)], peripartum	Third trimester, the validated food frequency questionnaire	0 g/wk: 169 (43%) 0–340 g/wk: 178 (46%) ≥ 340 g/wk: 44 (11%)	BSID-II: PDI; 12 mo; *n* = 270	Significant negative association between Pb measured in maternal blood and motor development at 12 mo, β (95% CI): −11.0 (−21, −1.2), *P* < 0.05	Nonsignificant, positive association with motor development if seafood intake ≥ twice/wk, β (95% CI): 0/wk: ref. 0 < ingestion < twice/wk: 0.38 (−2.5, 3.3) Ingestion ≥ twice/wk: 2.2 (−2.6, 6.9)
Rothenberg, 2021 [22] Daxin County cohort; China				BSID-II: PDI; 36 mo; *n* = 190	No association between Pb measured in maternal blood and motor development at 12 mo and 36 mo, β (95% CI): –0.95 (−3.3, 1.4), *P* > 0.05	Significant, positive association with motor development if seafood intake ≥2 svg/wk, β (95% CI): 0 svg/wk: ref. 0 < svg/wk <2: 0.89 (−1.4, 3.2), *P* > 0.05 ≥2 svg/wk: 4.0 (0.23, 7.7), *P* < 0.05
Behavioral problems						
Tatsuta, 2012 [25] Tohoku study of child development; Japan	Maternal cord blood median (5th–95th percentile) [1.0 *μ*g/dL (0.5, 1.7)], immediately after birth	Fourth day after delivery, the food frequency questionnaire	Median^2^ (q1, q3): 397 g/wk (103.6, 974.2)	Japanese version of CBCL; 30 mo; *n* = 599	No association between Pb(log_10_) and CBCL subdomains. Internalizing behaviors: β: −0.11, *P* > 0.05 Externalizing behaviors: β: −0.04, *P* > 0.05 Total behavioral problems: β: −0.10, *P* > 0.05	No association between fish intake and CBCL subdomains Internalizing behaviors: β: −0.06, *P* > 0.05 Externalizing behaviors: β: −0.08, *P* > 0.05 Total behavioral problems: β: −0.07, *P* > 0.05

Abbreviations: BSID-II, Bayley scales of infant development; CBCL, child behavior checklist; CI, confidence interval; IQ, Intelligence quotient; K-WPPSI, Korean version of the Wechsler preschool and primary scale of intelligence, revised edition; MDI, mental developmental index; min, max, minimum, maximum; NHANES, national health and nutrition examination survey; NR, not reported; Pb, lead; PDI, psychomotor developmental index; ref, reference category; q1, first quartile; q3, third quartile; SD, standard deviation; svg, serving.

1 Units were converted into grams/week for comparison across studies.

2 Converted from 20.7 kg/y using NHANES-2013 [42] estimate of 4.6 serving/mo and serving size =170 g.

### Pb exposure during PL

Exposure to Pb was assessed in maternal blood during late pregnancy or at delivery [21,22,26] or in cord blood immediately after birth [25]. The median Pb concentrations were 1.0 *μ*g/dL (5th –95th percentile: 0.5, 1.7) in the Japanese cohort [25] and 2.6 *μ*g/dL (minimum, maximum: 1.1, 7.8) in the Chinese cohort. The article, based on a Korean cohort with the lowest levels of seafood intake, did not report the maternal Pb concentrations [26].

### Neurodevelopmental outcomes

Neurodevelopmental outcomes in the included articles were cognitive development [21,22,26], motor development [21,22], and behavioral problems [25] among the children. Cognitive developmental outcomes were assessed using the adapted versions of Wechsler preschool and primary scale of intelligence at age 60 mo in 1 study [26] and Bayley scales of infant development-II at ages 12 and 36 mo [21,22]. Motor development was assessed in 2 studies using Bayley scales of infant development-II at ages 12 and 36 mo [21,22]. Children’s internalizing and externalizing behavioral problems, as well as total behavioral problems, were assessed using the Japanese version of the child behavior checklist at age 30 mo [25].

### Pb and child neurodevelopment

#### Pb and cognition

Three articles from 2 studies assessed the association between Pb and cognitive development [21,22,26]. Of those, 1 prospective cohort study assessed cognition in children at 12 and 36 mo [21,22]. At 12 mo, there was a nonsignificant negative association between maternal Pb concentrations measured peripartum and cognitive development [21]. When child assessment scores at 12 and 36 mo were included in the analysis [22], there was no association between maternal Pb concentrations and child cognitive development. The other study stated, there was not a significant association between maternal Pb concentrations during pregnancy and Intelligence quotient (IQ) in 5-y-old children but did not report the direction or size of the effect [26]. Overall, these findings do not support an association between Pb concentrations assessed during pregnancy and child cognitive development (Table 1).

#### Pb and motor development

Two articles from 1 prospective cohort study [21,22] reported the relationship between prenatal exposure to Pb and motor development (Table 1). A significant negative association was found between maternal Pb concentrations and motor scores at 12 mo [21]. However, the magnitude of the estimate was attenuated and no longer significant at the 36-mo follow-up [22]. This evidence suggests there may be an inverse association between maternal Pb concentrations and infant motor outcomes, but the association attenuates over time.

#### Pb and behavioral outcomes

One study examined maternal Pb concentrations and behavioral problems among 30 mo old children [25] and found no associations between maternal Pb and internalizing and externalizing behavioral problems or total behavioral problems in children (−0.04 ≤ β ≤−0.11, *P* > 0.05) (Table 1).

### Seafood intake and neurodevelopment

Higher maternal seafood consumption during pregnancy was significantly associated with higher cognitive and motor development scores at 12 mo (β: 4.1; 95% CI: 0.04, 8.2) and 36 mo in 1 prospective cohort study [β: 4.0; 95% CI: (0.23, 7.7), *P* < 0.05] [21,22]. Related to cognition, another study assessed the relationship between maternal seafood intake, by tertiles, and mean IQ scores in 5-y-old children [26]. There was little difference in verbal, performance, and total IQ scores across tertiles (Table 1). One study found no association between seafood intake and behavior scores (behavioral problems; r < −0.1, *P* > 0.05) measured at 30 mo old [25].

### RoB and GRADE assessment

All studies had RoB concerns. Table 2 [21,22,25,26] illustrates the domain-specific RoB assessments across the studies. Two articles were determined to be at high RoB overall [21,22], whereas 1 was rated as very high risk [26] and the other as “some concerns” for RoB [25]. The articles rated high or very high RoB had concerns due to confounding because they did not account for several key confounders (nondietary sources of PUFAs) and confounders used to measure socioeconomic status (Table 2) [21,22,26]. One article was at high RoB for several additional domains, including measurement of exposure, missing observations, and selective reporting of findings [26].

**TABLE 2:**
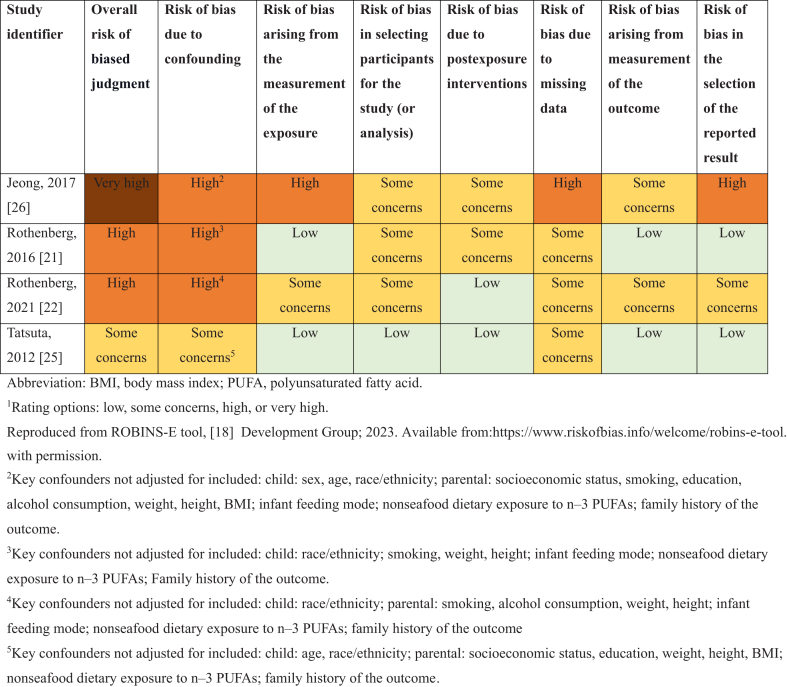
Risk of bias assessment.^1^

The certainty of evidence was rated very low for each neurodevelopment outcome due to lack of evidence and limitations in the evidence (Table 3) [21,22,25,26]. RoB was a concern stemming from confounding, exposure measurements, and/or missing data. Consistency was a concern because data were only available from 1 or 2 studies for each outcome, thus limiting the ability to assess inconsistency. Indirectness was downgraded because participants in the included studies (all from Asian countries) were not fully diverse and may not generalize to other population groups. Further, these studies were designed to measure mercury from seafood, and Pb was a secondary assessment/analysis. The evidence was imprecise, and given the lack of evidence, publication bias was a concern.

**TABLE 3:** Certainty of evidence using GRADE.^1^

*n* articles; study identifier	Risk of bias^2^	Inconsistency^3^^,^^4^	Indirectness^3^	Imprecision^3^^,^^5^	Publication bias^6^^,^^7^	Summary of findings^8^	Certainty^9^
Cognitive development^10^							
Three articles from 2 studies; Jeong 2017, [26] Rothenburg 2016, [21] Rothenburg 2021 [22]	Very serious; all studies rated high or very high risk of bias due to confounding, measurement of exposure, missing data, and selective reporting of findings	Very serious; mixed results for both seafood and lead associations with cognition across the 3 articles	Serious; not possible to determine that the Pb exposure is directly from seafood, and thus directness in terms of exposure is unclear; only included children 6 mo to 6 y; only included Asian populations	Serious; wide CI with a small total sample (*n* = ∼800)^11^	Strongly detected; only 2 studies	The evidence does not support an association between maternal exposure to Pb measured during pregnancy and cognitive development in the child at ages 1–6 y	Very low
Motor development^10^							
Two articles from 1 study; Rothenburg, 2016 [21] Rothenburg, 2021 [22]	Serious; rated as high RoB ratings based on confounding	Not applicable; only 1 study	Serious; not possible to determine that the Pb exposure is directly from seafood, and thus directness in terms of exposure is unclear; only included ages 12 – 36 mo; only included Asian populations	Serious; wide CI with a small sample (*n* = ∼270)^11^	Strongly detected; only 2 articles from 1 study	The evidence suggests an inverse association between maternal Pb measured during pregnancy and motor development in the child at ages 0–3 y	Very low
Behavioral problems^10^							
One article from 1 study; Tatsuta, 2012[25]	Not serious	Not applicable; only 1 study	Serious; not possible to determine that the Pb exposure is directly from seafood, and thus directness in terms of exposure is unclear; only included ages 2–6 y; only included Asian populations	Serious; there was no CI not reported (*n* = 306)^11^	Strongly detected; only 1 study	The evidence does not support an association between maternal Pb measured during pregnancy and behavioral problems in the child at ages 2–6 y	Very low

Abbreviations: CI, confidence interval; Pb, lead; RoB, risk of bias.

1 Grading of recommendations, assessment, development, and evaluation. Reproduced from H.J. Schünemann, C. Cuello, E.A. Akl, R.A. Mustafa, J.J. Meerpohl, K. Thayer, et al., GRADE guidelines: 18. How ROBINS-I and other tools to assess risk of bias in nonrandomized studies should be used to rate the certainty of a body of evidence, J Clin. Epidemiol. 111 (2019) 105–114, https://doi.org/10.1016/j.jclinepi.2018.01.012 with permission

2 Downgrading domain. Response options: not serious, serious, very serious, or extremely serious.

3 Downgrading domain. Response options: not serious, serious, or very serious.

4 Studies were rated as “severe” if there were <3 articles and “very severe” if there were <2 articles in a particular outcome domain.

5 All studies started as serious because all domains included null results, which could be an indicator of imprecision.

6 Downgrading domain. Response options: undetected or strongly detected.

7 If <3 articles were included, then publication bias was automatically strongly detected due to a lack of sufficient information to confidently rule out publication bias.

8 Large effect, plausible confounding, and dose-response domains are not shown in the table because these domains were either not assessed or were “No” for all outcomes and, thus, did not provide an opportunity to upgrade the evidence.

9 GRADE rating options: high, moderate, low, and very low.

10 All included studies were nonrandomized studies of exposure.

11 Total sample size is the sum of sample sizes across the contributing studies. The largest sample size was considered for counting the total sample size if there were multiple articles per study.

## Discussion

In this review, we investigated the evidence for the association between exposure to Pb from seafood during PL and neurodevelopmental outcomes in the child. This evidence does not support an association between Pb concentrations during pregnancy and cognitive development (2 cohort studies) or internalizing/externalizing behavior problems in children (1 cohort study). There may be an inverse association between maternal Pb concentrations and infant motor development; however, the association was attenuated over time. The evidence-based 1 prospective cohort study suggests that higher seafood consumption during pregnancy is associated with better cognition and motor development outcomes, a finding supported by a recent systematic review [27]. Only 1 article in the present evidence assessed the direct association between seafood intake and Pb concentrations, indicating weak and nonsignificant correlations [21].

Pb is known to be a toxic heavy metal that disrupts neurological development. Therefore, the lack of an association between Pb concentrations and child development in this evidence may be considered surprising, but there are several possible explanations. First, the timing of exposure assessment during pregnancy is particularly noteworthy. The half-life of Pb in the blood is relatively short (∼28 d, which can fluctuate due to factors such as route of exposure and particle size); therefore, assessments in late pregnancy pose a limitation when using late pregnancy measurements to assess cumulative exposure throughout gestation, or especially to assess exposure during early pregnancy, which may have greater implications for fetal neurodevelopment [[28], [29], [30], [31], [32]]. Although 1 study [33] found a stronger association between Pb exposure and adverse cognitive developmental outcomes when Pb was measured during the first trimester, focusing solely on early pregnancy may not adequately capture cumulative Pb exposure. Pb concentrations may increase in later pregnancy due to enhanced bone resorption, suggesting that repeated assessments of Pb at multiple time points, along with recording seafood intake during pregnancy, would provide a more comprehensive evaluation of exposure and its potential impact on neurodevelopment [34]. Second, the association between Pb and neurodevelopmental outcomes may be masked by co-exposures with other nutrients, such as PUFAs, or toxicants in seafood, such as arsenic, cadmium, and mercury, which could impact neurodevelopment (Supplemental Figure 2), or due to inadequate control for confounders such as socioeconomic status or prenatal supplementation.

Another possible explanation for not finding an association between Pb concentrations and child neurodevelopment outcomes could be that the Pb concentrations in the study participants were relatively low, such that they did not affect neurodevelopmental outcomes. The reported median Pb concentrations in the current evidence are 2.6 *μ*g/dL [minimum, maximum: (0.96, 7.8)] [21,22] and 1.0 *μ*g/dL (5th–95th percentile: 0.5–1.7) [25] (Table 1). Based on additional data analysis, the NASEM committee reported that meat, poultry, and fish combined only contributed to ∼3% of Pb exposure, and as such, Pb exposure from fish is lower than the interim reference level of 3 *μ*g/d [15]. Despite these relatively low values, previous studies documented inverse associations of median maternal blood Pb concentrations ≤5 *μ*g/dL [[35], [36], [37]] and as low as 1.29 *μ*g/dL [38] with cognitive development and school performance. Furthermore, organizations such as the Agency for Toxic Substances and Disease Registry indicated that maternal Pb exposure at any level could potentially affect child neurodevelopment without a discernible threshold for blood Pb concentrations [39,40].

Similar to the timing of the exposure assessment, the timing of the outcome assessment may also contribute to the absence of an association between maternal Pb and neurodevelopment outcomes. For example, the only statistically significant result found within this evidence was in the youngest age assessed, 12 mo. At 36-mo follow-up, the association was no longer significant. Other outcome assessments occurred at 30 mo and 60 mo. Neurodevelopmental differences in early infancy may attenuate as the child develops; further, at older ages, postnatal exposure to Pb and other substances may influence neurodevelopment and, therefore, should be considered in the analysis. However, more research measuring postnatal Pb exposures and outcomes in infancy with additional follow-up assessments to measure changes over time is needed to confirm this.

Finally, we cannot make a conclusion related to Pb from seafood because only 1 study in the current evidence base analyzed the relationship between maternal seafood intake and blood Pb concentrations, and they did not find a significant association (r < 0.1, *P* > 0.05) [21]. In the absence of a direct measurement of Pb in the seafood consumed, it is not possible to definitively assign seafood as the source of Pb exposure. The inclusion criteria applied to this systematic review, as well as the other reviews in the series for the NASEM committee, required that an article report data and analyses between maternal seafood intake, Pb exposure, and child neurodevelopment outcomes. Thus, a limitation of our analysis is that studies that may have measured this data but reported the relationships across multiple articles (i.e., not in a single article) would not have been included. This potential omission may be a source of bias. Although the inclusion criteria were designed to focus on Pb exposure from seafood, there are limitations related to methods used to assess Pb from seafood intake, specifically the use of FFQs. The questionnaires used to assess seafood intake in the included studies were administered at a single time point during pregnancy or immediately after delivery and did not capture detailed information on the types of seafood consumed. The limitations of FFQs in differentiating between various seafood types are notable, as different species accumulate Pb and other contaminants at varying rates and to different concentrations [41,42]. This lack of specificity of intake may have impacted observed associations between seafood intake and Pb concentrations. Furthermore, environmental sources of Pb exposure may contribute more significantly to total Pb exposure than seafood [15].

The populations in the included studies were from Asian countries, which may not be generalizable to the United States, particularly if seafood intake and Pb exposure differ between these populations. Seafood intake in the pregnant United States population (median 182 g/wk) [43] is comparable to the Chinese cohort (46% consumed 0–340 g/wk of seafood) and lower than the Japanese cohort (median 397g/wk). Further, maternal median Pb concentrations during pregnancy in the United States (0.44 *μ*g/dL) are lower than maternal Pb concentrations reported in the evidence in these Asian populations (median concentrations of 2.6 and 1.0 *μ*g/dL) [21,22,25]. Although there are other population differences that may impact generalizability based on seafood intake and Pb exposure alone, we would not necessarily expect to find an association in a United States-based cohort.

Our systematic review adhered to all PRISMA reporting criteria followed a well-vetted protocol, received input from a NASEM expert committee, and was conducted by an independent third-party research team to reduce bias. There were limitations in the evidence, including RoB in the studies included and potential bias from the conservative inclusion criteria. The certainty of evidence was very low due to several limitations in the evidence, mainly due to the limited number of studies, all with some concerns, high or very high RoB. Because of the considerable heterogeneity, particularly related to outcome assessment and statistical methods used in the studies included, we could not conduct a pooled analysis. Additionally, as the studies were conducted in Asian populations, the findings are not necessarily generalizable to the United States population.

Additional studies in more diverse populations measuring maternal Pb in early pregnancy, Pb concentrations in breast milk, and child outcomes in early infancy with additional follow-up assessments are needed to determine with greater certainty if seafood consumption during PL is a source of concern regarding Pb exposure and child neurodevelopment.

## Conclusion

This review offers a valuable contribution by investigating the potential links between Pb and seafood exposure during PL and neurodevelopmental outcomes in children and underscores a critical gap in the literature on the relationship between Pb exposure from seafood during PL and child development.

## Conflict of interest

The authors report no conflicts of interest.
