# Supplementary material for: Association between Maternal Lead Exposure from Seafood Consumption and Neurodevelopment: A Systematic Review
Source: Adv Nutr. 2025 Jan 21;16(3):100380. doi: 10.1016/j.advnut.2025.100380 (PMC11875176; doi:10.1016/j.advnut.2025.100380)
Supplement: Multimedia component 1 [file mmc1.pdf]

Association Between Maternal Lead Exposure from Seafood Consumption and Neurodevelopment: A Systematic Review

Arin A. Balalian

Table of Contents

Supplementary Table 1a. PRISMA 2020 Main Checklist 2

Supplementary Table 1b. PRIMSA Abstract Checklist 7

Supplementary Table 2. Search Strategy and Search Strings 9

Supplementary Table 3: Inclusion and exclusion criteria for a systematic review of the association between perinatal exposure to lead from seafood consumption and child neurodevelopment 31

Supplementary Figure 1. Eligible articles for the systematic review based on the investigated relationships between exposures and outcomes 33

Supplementary Figure 2. Mechanism of co-pollutant and other nutrients confounding 34

Supplemental Online Content

Supplementary Table 1a. PRISMA 2020 Main Checklist

Topic	No.	Item	Location where item is reported
**TITLE**			
**Title**	1	Identify the report as a systematic review.	page 1
**ABSTRACT**			
**Abstract**	2	See the PRISMA 2020 for Abstracts checklist.	
**INTRODUCTION**			
**Rationale**	4	Describe the rationale for the review in the context of existing knowledge.	page 4
**Objectives**	4	Provide an explicit statement of the objective(s) or question(s) the review addresses.	page 5
**METHODS**			
**Eligibility criteria**	5	Specify the inclusion and exclusion criteria for the review and how studies were grouped for the syntheses.	page 5,6
**Information sources**	6	Specify all databases, registers, websites, organizations, reference lists and other sources searched or consulted to identify studies. Specify the date when each source was last searched or consulted.	page 5
**Search strategy**	7	Present the full search strategies for all databases, registers and websites, including any filters and limits used.	Supplementary Table 2
**Selection process**	8	Specify the methods used to decide whether a study met the inclusion criteria of the review, including how many reviewers screened each record and each report retrieved, whether they worked independently, and if applicable, details of automation tools used in the process.	page 5

Topic	No.	Item	Location where item is reported
**Data collection process**	9	Specify the methods used to collect data from reports, including how many reviewers collected data from each report, whether they worked independently, any processes for obtaining or confirming data from study investigators, and if applicable, details of automation tools used in the process.	page 6
**Data items**	10a	List and define all outcomes for which data were sought. Specify whether all results that were compatible with each outcome domain in each study were sought (e.g., for all measures, time points, analyses), and if not, the methods used to decide which results to collect.	page 6
	10b	List and define all other variables for which data were sought (e.g., participant and intervention characteristics, funding sources). Describe any assumptions made about any missing or unclear information.	Supplementary Table 3
**Study risk of bias assessment**	11	Specify the methods used to assess risk of bias in the included studies, including details of the tool(s) used, how many reviewers assessed each study and whether they worked independently, and if applicable, details of automation tools used in the process.	Page 6-7
**Effect measures**	12	Specify for each outcome the effect measure(s) (e.g., risk ratio, mean difference) used in the synthesis or presentation of results.	Table 1
**Synthesis methods**	13a	Describe the processes used to decide which studies were eligible for each synthesis (e.g., tabulating the study intervention characteristics and comparing against the planned groups for each synthesis (item 5)).	Page 7
	13b	Describe any methods required to prepare the data for presentation or synthesis, such as handling of missing summary statistics, or data conversions.	Page 7
	13c	Describe any methods used to tabulate or visually display results of individual studies and syntheses.	Page 7

Topic	No.	Item	Location where item is reported
	13d	Describe any methods used to synthesize results and provide a rationale for the choice(s). If meta-analysis was performed, describe the model(s), method(s) to identify the presence and extent of statistical heterogeneity, and software package(s) used.	Page 7
	13e	Describe any methods used to explore possible causes of heterogeneity among study results (e.g., subgroup analysis, meta-regression).	N/A
	13f	Describe any sensitivity analyses conducted to assess robustness of the synthesized results.	N/A
**Reporting bias assessment**	14	Describe any methods used to assess risk of bias due to missing results in a synthesis (arising from reporting biases).	Page 6
**Certainty assessment**	15	Describe any methods used to assess certainty (or confidence) in the body of evidence for an outcome.	Page 7
**RESULTS**			
**Study selection**	16a	Describe the results of the search and selection process, from the number of records identified in the search to the number of studies included in the review, ideally using a flow diagram.	Figure 1
	16b	Cite studies that might appear to meet the inclusion criteria, but which were excluded, and explain why they were excluded.	N/A
**Study characteristics**	17	Cite each included study and present its characteristics.	Table 1
**Risk of bias in studies**	18	Present assessments of risk of bias for each included study.	Table 2

Topic	No.	Item	Location where item is reported
**Results of individual studies**	19	For all outcomes, present, for each study: (a) summary statistics for each group (where appropriate) and (b) an effect estimates and its precision (e.g., confidence/credible interval), ideally using structured tables or plots.	Table 1
**Results of syntheses**	20a	For each synthesis, briefly summarize the characteristics and risk of bias among contributing studies.	Table 1
	20b	Present results of all statistical syntheses conducted. If meta-analysis was done, present for each the summary estimate and its precision (e.g., confidence/credible interval) and measures of statistical heterogeneity. If comparing groups, describe the direction of the effect.	N/A
	20c	Present results of all investigations of possible causes of heterogeneity among study results.	N/A
	20d	Present results of all sensitivity analyses conducted to assess the robustness of the synthesized results.	N/A
**Reporting biases**	21	Present assessments of risk of bias due to missing results (arising from reporting biases) for each synthesis assessed.	Table 2
**Certainty of evidence**	22	Present assessments of certainty (or confidence) in the body of evidence for each outcome assessed.	Table 3
**DISCUSSION**			
**Discussion**	23a	Provide a general interpretation of the results in the context of other evidence.	page 11-12
	23b	Discuss any limitations of the evidence included in the review.	pages 12-14
	23c	Discuss any limitations of the review processes used.	Pages14-15
	23d	Discuss implications of the results for practice, policy, and future research.	pages 15

Topic	No.	Item	Location where item is reported
**OTHER INFORMATION**			
**Registration and protocol**	24a	Provide registration information for the review, including register name and registration number, or state that the review was not registered.	Page 5
	24b	Indicate where the review protocol can be accessed, or state that a protocol was not prepared.	Page 5
	24c	Describe and explain any amendments to information provided at registration or in the protocol.	Page 5
**Support**	25	Describe sources of financial or non-financial support for the review, and the role of the funders or sponsors in the review.	Page 16
**Competing interests**	26	Declare any competing interests of review authors.	Page 16
**Availability of data, code, and other materials**	27	Report which of the following are publicly available and where they can be found template data collection forms; data extracted from included studies; data used for all analyses; analytic code; any other materials used in the review.	Page 16

Supplementary Table 1b. PRIMSA Abstract Checklist

Topic	No.	Item	Reported?
**TITLE**			
**Title**	1	Identify the report as a systematic review.	Yes
**BACKGROUND**			
**Objectives**	2	Provide an explicit statement of the main objective(s) or question(s) the review addresses.	Yes
**METHODS**			
**Eligibility criteria**	3	Specify the inclusion and exclusion criteria for the review.	Yes
**Information sources**	4	Specify the information sources (e.g., databases, registers) used to identify studies and the date when each was last searched.	Yes
**Risk of bias**	5	Specify the methods used to assess risk of bias in the included studies.	Yes
**Synthesis of results**	6	Specify the methods used to present and synthesize results.	Yes
**RESULTS**			
**Included studies**	7	Give the total number of included studies and participants and summarize relevant characteristics of studies.	Yes
**Synthesis of results**	8	Present results for main outcomes, preferably indicating the number of included studies and participants for each. If meta-analysis was done, report the summary estimate and confidence/credible interval. If	Yes

Topic	No.	Item	Reported?
		comparing groups, indicate the direction of the effect (i.e., which group is favored).	
**DISCUSSION**			
**Limitations of evidence**	9	Provide a brief summary of the limitations of the evidence included in the review (e.g., study risk of bias, inconsistency, and imprecision).	Yes
**Interpretation**	10	Provide a general interpretation of the results and important implications.	Yes
**OTHER**			
**Funding**	11	Specify the primary source of funding for the review.	Yes
**Registration**	12	Provide the register name and registration number.	No

From: Page MJ, McKenzie JE, Bossuyt PM, Boutron I, Hoffmann TC, Mulrow CD, et al. The PRISMA 2020 statement: an updated guideline for reporting systematic reviews. MetaArXiv. 2020, September 14. DOI: 10.31222/osf.io/v7gm2. For more information, visit: www.prisma-statement.org

Supplementary Table 2. Search Strategy and Search Strings

MEDLINE
Database: MEDLINE
Platform: Ovid
Date of search: October 10, 2024
Limits: Not animal studies

Concept	Line	Search Strategy
seafoods	1	exp Fishes/ or exp Fish Proteins/ or Shellfish Proteins/ or exp Seafood/ or exp Fish Products/ or exp Fishes/ or exp Fish Proteins/ or Shellfish Proteins/ or exp Seafood/ or exp Fish Products/ or exp Crustacea/ or exp Mollusca/ or exp Cetacea/ or (seafood* or "sea food*" or ((fish or fishes or marine) and (food* or diet or diets or dieta* or diete* or dieti* or eat* or ingest* or nutrit* or nutrient? or cuisine* or culinary or consum* or protein? or product? or meal)) or ((fish or fishes or marine) adj3 (consum* or protein? or product? or meal or flour or fatty)) or fishmeat? or abalone* or aholehole* or ailia* or alewife* or alfonsino* or amberjack* or anchov* or angelfish* or "angel fish*" or arkshell* or "ark shell*" or armorhead* or "armor head*" or arowana* or ayre* or bacha? or bagrid? or bangamar* or (barb and (fish or fishes)) or barnacle* or barracouta* or barracuda* or barramundi* or basa or bass or basses or bata or beardfish* or "beard fish*" or bigeye* or (bittersweet? and mollusc?) or blackfish* or "black fish*" or bluefish* or "black fish*" or bluegill* or "blue gill*" or bluenose* or "blue nose*" or boarfish* or "boar fish*" or bocachico* or boga? or bogue* or bonefish* or "bone fish*" or bonito* or bonnethead* or "bonnet head*" or bonnetmouth* or "bonnet mouth*" or bowfin* or "bow fin*" or bream? or brotula* or buffalofish* or "buffalo fish*" or bullhead? or "bull head?" or (bumper? and (fish or fishes)) or burbot* or butterfish* or "butter* fish*" or butterflyfish* or caballa* or cabrilla* or cachama* or caiman* or calamari* or calbasu* or caparari* or capelin* or capensis* or carate* or cardinalfish* or "cardinal fish*" or carp or carplet* or cascarudo* or catalina* or catfish* or "cat fish*" or catla* or chanda* or channa* or (char? and (fish or fishes)) or (char? adj1 (arctic or alsatian or common or alpine or oregon)) or charal* or chimaera* or chiring* or (chub? and (fish or fishes)) or cichlid? or cisco* or clam or clams or claresse* or clarias* or cobia* or cockle? or cod or conch or conches or coquina* or corbina* or cornetfish* or "cornet fish*" or coroata* or corvina* or cottonwick* or "cotton wick*" or cowfish* or "cow fish*" or cra#fish* or "cra# fish*" or crab? or crabmeat* or crappie* or crevalle* or croaker* or crustacean* or cubbyu* or cuirass or cuirasses or cunner* or curimbata* or cusk? or cuskeel* or cutlassfish* or "cutlass fish*" or cuttlefish* or "cuttle fish*" or (dab? and (fish or fishes)) or dace or damselfish* or "damsel fish*" or dogcockle* or dorab* or dories or dory? or driftfish* or "drift fish*" or drum or drummer* or duckbill* or "duck bill*" or eel? or eelpout* or em?r#ldperch* or escargot* or escolar* or fanfish* or "fan fish*" or featherback* or "feather back*" or fiddler* or filefish* or "file fish*" or flathead* or "flat head*" or flatwhiskered* or "flat whiskered*" or flounder? or (fluke? and (fish or fishes)) or flyingfish* or "flying fish*" or fugu or fusilier* or gafftopsail* or (gag? and (fish or fishes)) or gar or garfish* or "gar fish*" or gemfish* or "gem fish*" or gilleybaka* or goatfish* or "goat fish*" or gobies or goby or goldeye* or "gold eye*" or goonch* or goosefish* or "goose fish*" or goram* or gourami* or grayling* or greeneye* or greenling* or grenadier* or grouper* or grunion* or (grunt? and (fish or fishes)) or grunter* or guitarfish* or "guitar fish*" or gurnard* or haddock* or hagfish* or "hag fish*" or hake?

		or halfbeaks* or "half beak*" or halfmoon? or "half moon*" or halibut* or hamlet* or hammerhead* or "hammer head*" or hassar* or hatchetfish* or "hatchet fish*" or hawkfish* or "hawk fish*" or herring? or highwaterman* or hilsa? or (hind? and (fish or fishes)) or hogfish* or "hog fish*" or houndfish* or "hound fish*" or hualca* or icefish* or "ice fish*" or inaha* or isopod* or jellyfish* or "jelly fish*" or jewfish* or "jew fish*" or jobfish* or "job fish*" or kahawai* or kajuli* or kanpachi* or kelpfish* or "kelp fish*" or killifish* or "killi fish*" or kingfish* or "king fish*" or kingklip* or "king klip*" or knifefish* or "knife fish*" or knifejaw* or "knife jaw*" or krill? or ladyfish* or "lady fish*" or lancet* or langostino* or leaffish* or "leaf fish*" or leatherjacket* or "leather jacket*" or limpet* or (ling? and (fish or fishes)) or lingcod* or lionfish* or "lion fish*" or lizardfish* or "lizard fish*" or loach* or lobster* or louvar* or lumpfish* or "lump fish*" or lyretail* or "lyre tail*" or mackerel* or manta? or margate* or marlin? or megrim* or menhaden* or merex* or miiuy* or milkfish* or "milk fish*" or minnow* or moga? or mojarra* or moki? or mola or molas or monkfish* or "monk fish*" or mooneye* or "moon eye*" or moonfish* or "moon fish*" or moonsnail* or "moon snail*" or morwong* or mrigal* or mullet? or murex or murexes or muskellunge* or mussel? or mystus or needlefish* or "needle fish*" or nodoguro* or noodlefish* or "noodle fish*" or octopus* or octopi or oilfish* or "oil fish*" or opah? or opaleye* or "opal eye*" or oscar? or oyster? or pabdah* or packoo* or pacu? or paddlefish* or "paddle fish*" or paima? or pangasius* or pargo? or parrotfish* or "parrot fish*" or pearlspot* or "pearl spot*" or penshell* or "pen shell*" or perch or perches or periwinkle? or picarel* or pickerel* or pike? or pikeperch* or pilchard* or pintado* or pipefish* or "pipe fish*" or piramutaba* or pirarucu* or plaice* or pleco? or pollock? or pomfret? or pompanito* or pompano* or ponyfish* or "pony fish*" or porgy? or porgies or porkfish* or "pork fish*" or potasi? or pout? or prawn? or puffer* or punti? or quahog* or queenfish* or "queen fish*" or (racehorse? and (fish or fishes)) or ((ray or rays) and (fish or fishes)) or redfish* or "red fish*" or redhorse* or "red horse*" or remora? or rita? or rockfish* or "rock fish*" or rockling* or rohu? or roosterfish* or "rooster fish*" or rosefish* or "rose fish*" or roughback* or "rough back*" or roughies or roughy* or ruff or (runner? and (fish or fishes)) or sablefish* or "sable fish*" or sailfish* or "sail fish*" or salmon* or sampa? or sanddab* or sandeel* or (sander? and (fish or fishes)) or sandfish* or "sand fish*" or sandperch* or sardine? or sargo? or sauger? or sauries or saury* or sawfish* or "saw fish*" or scad? or scallop? or scamp? or schoolmaster* or scorpionfish* or "scorpion fish*" or scup or scups or scupper? or seabass* or seabob* or "sea bob*" or seabream* or searobin* or "sea robin*" or seasnail* or "sea snail*" or shad or shads or shadefish* or "shade fish*" or shark? or sharksucker? or sheatfish* or "sheat fish*" or sheep?head? or "sheep? head?" or shellfish* or "shell fish*" or shiner? or shrimp? or sild or silds or sillago* or silverside* or "silver side*" or skate? or skilfish* or "skil fish*" or slipmouth* or "slip mouth*" or slipperysnail* or "slippery snail*" or (smelt* and (fish or fishes)) or smoothhound* or "smooth hound*" or snail? or snakehead? or snapper? or snook? or soldierfish* or "soldier fish*" or (sole* and (fish or fishes)) or s?rubi? or spadefish* or "spade fish*" or spearfish* or "spear fish*" or spinef##t* or "spine f##t*" or (spot* and (fish or fishes)) or sprat or sprats or squid? or squirrelfish* or "squirrel fish*" or stargazer* or "star gazer*" or (stinging and (fish or fishes)) or stingray* or "sting ray*" or straptail* or "strap tail*" or sturgeon* or (sucker? and (fish or fishes)) or suckerfish* or sunfish* or "sun fish*" or surfclam* or "surf clam*" or surfperch* or surgeonfish* or "surgeon fish*" or sutchi? or swai? or sweetlip* or "sweet lip*" or swordfish* or "sword fish*" or tambaqui* or tang or tangs or tarakihi* or tarpon? or tautog* or tench or tenches or therapon? or thornyhead* or "thorny head*" or threadfin* or "thread fin*" or tigerfish* or "tiger fish*" or

		tigerperch* or tilapia* or tilefish* or "tile fish*" or (tinfoil* and (fish or fishes)) or toadfish* or "toad fish*" or tomcod* or tomtate* or to?ng?esole* or "to?ng?e sole*" or toothfish* or "tooth fish*" or (torpedo* and (fish or fishes)) or tra or tras or trevallies or trevally* or triggerfish* or "trigger fish*" or tripletail* or "tripe tail*" or trout? or trumpeter* or trumpetfish* or "trumpet fish*" or trunkfish* or "trunk fish*" or tullibee* or tuna or tunas or tunicata* or turbot? or turtle? or unicornfish* or "unicorn fish*" or urchin? or vendace* or volute? or wahoo* or walleye* or "wall eye*" or warehou or weakfish* or "weak fish*" or weever* or whale? or whalemeat? or whelk? or whiff? or whiskerfish* or "whisker fish*" or whitefish* or "white fish*" or whiting* or wolffish* or "wolf fish*" or wrasse? or yellowtail* or "yellow tail*" or zander? or zungaro* or "lau lau*" or "mahi mahi*" or "sergeant major*" or "sea cucumber*" or "american smelt*" or "bombay duck*" or "irish lord*" or "pen shell*" or "salmo salar" or "sand lance?" or "spoon worm*" or "striped dwarf*" or "top shell*" or gadiform* or perciform* or decapodiform* or sushi? or sashimi? or ce#iche or se#iche or gravlax or carpaccio or crudo or poke or hinava or "gohu ikan" or esqueixada or kelaguen or namero or kilawin or stroganina or yusheng or "yee sang" or "yuu sahng" or "lo sahng" or koi or kokoda or kuai or lakerda or "larb pla" or "ota ika" or tiradito or xato or umai).ti,ab,kf.
pregnancy	2	exp Pregnancy/ or exp Pregnancy Complications/ or exp Pregnancy Outcome/ or (pregnan* or prenatal* or "post natal*" or postnatal* or "post natal*" or antenatal* or "ante natal*" or perinatal* or "peri natal*" or postpartum or "post partum" or f?etus* or f?etal or maternal or gravid* or birth*).ti,ab,kf,hw.
lactation	3	exp Lactation/ or exp Breast Feeding/ or exp Milk, Human/ or (lactat* or breastfe* or (breast adj (fed or feed*)) or ((human? or breast* or maternal or mother*) adj2 milk*) or breastmilk? or colostrum).ti,ab,kf.
infant	4	exp Infant/ or ((("0" or "1" or "2" or zero or one or two) adj5 (age? or year? or old)) or (("0" or "1" or "2" or zero or one or two) adj5 (yr or yrs or y or ys)) or ((month? or mnth? or mth? or ms or m or week? or wks or wk or w or ws or day? or ds or d) adj5 (old or age?)) or infan* or baby or babies or newborn* or "new born*" or neonat* or "neo nat*").ti,ab,kf.
	5	or/2-4
lead	6	exp Lead Poisoning/ or exp Lead/ or exp Lead Radioisotopes/ or exp Tetraethyl Lead/ or lead.nm,kf. or (pb or 208Pb or organolead* or plumbum or plumbic* or plumbate* or plumbous or tetraethyllead or (lead adj8 ("208" or metal* or organometal* or cation* or ion* or isotop* or radioisotop* or element* or microelement* or organic or inorganic or ore or blood* or chemical? or poison* or contam*))).ti,ab,kf.
search structure	7	1 and 5 and 6
not humans	8	(exp Animals/ not (exp Animals/ and Humans/)) or exp Animal Experimentation/ or exp Models, Animal/ or (mouse or mice or rat or rats or monkey or monkeys or "preclinical study").ti.
not not humans	9	7 not 8

Embase
Database: Embase
Platform: Ovid
Date of search: October 10, 2024
Limits: Not animal studies

Concept	Line	Search Strategy
seafoods	1	exp fish/ or exp sea food/ or fish consumption/ or fish protein/ or fish oil/ or contaminated fish/ or contaminated shellfish/ or contaminated mussel/ or exp Crustacea/ or exp Cetacea/ or exp mollusc/ or (seafood* or "sea food*" or ((fish or fishes or marine) and (food* or diet or diets or dieta* or diete* or dieti* or eat* or ingest* or nutrit* or nutrient? or cuisine* or culinary or consum* or protein? or product? or meal)) or ((fish or fishes or marine) adj3 (consum* or protein? or product? or meal or flour or fatty)) or fishmeat? or abalone* or aholehole* or ailia* or alewife* or alfonsino* or amberjack* or anchov* or angelfish* or "angel fish*" or arkshell* or "ark shell*" or armorhead* or "armor head*" or arowana* or ayre* or bacha? or bagrid? or bangamar* or (barb and (fish or fishes)) or barnacle* or barracouta* or barracuda* or barramundi* or basa or bass or basses or bata or beardfish* or "beard fish*" or bigeye* or (bittersweet? and mollusc?) or blackfish* or "black fish*" or bluefish* or "black fish*" or bluegill* or "blue gill*" or bluenose* or "blue nose*" or boarfish* or "boar fish*" or bocachico* or boga? or bogue* or bonefish* or "bone fish*" or bonito* or bonnethead* or "bonnet head*" or bonnetmouth* or "bonnet mouth*" or bowfin* or "bow fin*" or bream? or brotula* or buffalofish* or "buffalo fish*" or bullhead? or "bull head?" or (bumper? and (fish or fishes)) or burbot* or butterfish* or "butter* fish*" or butterflyfish* or caballa* or cabrilla* or cachama* or caiman* or calamari* or calbasu* or caparari* or capelin* or capensis* or carate* or cardinalfish* or "cardinal fish*" or carp or carplet* or cascarudo* or catalina* or catfish* or "cat fish*" or catla* or chanda* or channa* or (char? and (fish or fishes)) or (char? adj1 (arctic or alsatian or common or alpine or oregon)) or charal* or chimaera* or chiring* or (chub? and (fish or fishes)) or cichlid? or cisco* or clam or clams or claresse* or clarias* or cobia* or cockle? or cod or conch or conches or coquina* or corbina* or cornetfish* or "cornet fish*" or coroata* or corvina* or cottonwick* or "cotton wick*" or cowfish* or "cow fish*" or cra#fish* or "cra# fish*" or crab? or crabmeat* or crappie* or crevalle* or croaker* or crustacean* or cubbyu* or cuirass or cuirasses or cunner* or curimbata* or cusk? or cuskeel* or cutlassfish* or "cutlass fish*" or cuttlefish* or "cuttle fish*" or (dab? and (fish or fishes)) or dace or damselfish* or "damsel fish*" or dogcockle* or dorab* or dories or dory? or driftfish* or "drift fish*" or drum or drummer* or duckbill* or "duck bill*" or eel? or eelpout* or em?r#ldperch* or escargot* or escolar* or fanfish* or "fan fish*" or featherback* or "feather back*" or fiddler* or filefish* or "file fish*" or flathead* or "flat head*" or flatwhiskered* or "flat whiskered*" or flounder? or (fluke? and (fish or fishes)) or flyingfish* or "flying fish*" or fugu or fusilier* or gafftopsail* or (gag? and (fish or fishes)) or gar or garfish* or "gar fish*" or gemfish* or "gem fish*" or gilleybaka* or goatfish* or "goat fish*" or gobies or goby or goldeye* or "gold eye*" or goonch* or goosefish* or "goose fish*" or goram* or gourami* or grayling* or greeneye* or greenling* or grenadier* or grouper* or grunion* or (grunt? and (fish or fishes)) or grunter* or

		guitarfish* or "guitar fish*" or gurnard* or haddock* or hagfish* or "hag fish*" or hake? or halfbeaks* or "half beak*" or halfmoon? or "half moon*" or halibut* or hamlet* or hammerhead* or "hammer head*" or hassar* or hatchetfish* or "hatchet fish*" or hawkfish* or "hawk fish*" or herring? or highwaterman* or hilsa? or (hind? and (fish or fishes)) or hogfish* or "hog fish*" or houndfish* or "hound fish*" or hualca* or icefish* or "ice fish*" or inaha* or isopod* or jellyfish* or "jelly fish*" or jewfish* or "jew fish*" or jobfish* or "job fish*" or kahawai* or kajuli* or kanpachi* or kelpfish* or "kelp fish*" or killifish* or "killi fish*" or kingfish* or "king fish*" or kingklip* or "king klip*" or knifefish* or "knife fish*" or knifejaw* or "knife jaw*" or krill? or ladyfish* or "lady fish*" or lancet* or langostino* or leaffish* or "leaf fish*" or leatherjacket* or "leather jacket*" or limpet* or (ling? and (fish or fishes)) or lingcod* or lionfish* or "lion fish*" or lizardfish* or "lizard fish*" or loach* or lobster* or louvar* or lumpfish* or "lump fish*" or lyretail* or "lyre tail*" or mackerel* or manta? or margate* or marlin? or megrim* or menhaden* or merex* or miiuy* or milkfish* or "milk fish*" or minnow* or moga? or mojarra* or moki? or mola or molas or monkfish* or "monk fish*" or mooneye* or "moon eye*" or moonfish* or "moon fish*" or moonsnail* or "moon snail*" or morwong* or mrigal* or mullet? or murex or murexes or muskellunge* or mussel? or mystus or needlefish* or "needle fish*" or nodoguro* or noodlefish* or "noodle fish*" or octopus* or octopi or oilfish* or "oil fish*" or opah? or opaleye* or "opal eye*" or oscar? or oyster? or pabdah* or packoo* or pacu? or paddlefish* or "paddle fish*" or paima? or pangasius* or pargo? or parrotfish* or "parrot fish*" or pearlspot* or "pearl spot*" or penshell* or "pen shell*" or perch or perches or periwinkle? or picarel* or pickerel* or pike? or pikeperch* or pilchard* or pintado* or pipefish* or "pipe fish*" or piramutaba* or pirarucu* or plaice* or pleco? or pollock? or pomfret? or pompanito* or pompano* or ponyfish* or "pony fish*" or porgy? or porgies or porkfish* or "pork fish*" or potasi? or pout? or prawn? or puffer* or punti? or quahog* or queenfish* or "queen fish*" or (racehorse? and (fish or fishes)) or ((ray or rays) and (fish or fishes)) or redfish* or "red fish*" or redhorse* or "red horse*" or remora? or rita? or rockfish* or "rock fish*" or rockling* or rohu? or roosterfish* or "rooster fish*" or rosefish* or "rose fish*" or roughback* or "rough back*" or roughies or roughy* or ruff or (runner? and (fish or fishes)) or sablefish* or "sable fish*" or sailfish* or "sail fish*" or salmon* or sampa? or sanddab* or sandeel* or (sander? and (fish or fishes)) or sandfish* or "sand fish*" or sandperch* or sardine? or sargo? or sauger? or sauries or saury* or sawfish* or "saw fish*" or scad? or scallop? or scamp? or schoolmaster* or scorpionfish* or "scorpion fish*" or scup or scups or scupper? or seabass* or seabob* or "sea bob*" or seabream* or searobin* or "sea robin*" or seasnail* or "sea snail*" or shad or shads or shadefish* or "shade fish*" or shark? or sharksucker? or sheatfish* or "sheat fish*" or sheep?head? or "sheep? head?" or shellfish* or "shell fish*" or shiner? or shrimp? or sild or silds or sillago* or silverside* or "silver side*" or skate? or skilfish* or "skil fish*" or slipmouth* or "slip mouth*" or slipperysnail* or "slippery snail*" or (smelt* and (fish or fishes)) or smoothhound* or "smooth hound*" or snail? or snakehead? or snapper? or snook? or soldierfish* or "soldier fish*" or (sole* and (fish or fishes)) or s?rubi? or spadefish* or "spade fish*" or spearfish* or "spear fish*" or spinef##t* or "spine f##t*" or (spot* and (fish or fishes)) or sprat or sprats or squid? or squirrelfish* or "squirrel fish*" or stargazer* or "star gazer*" or (stinging and (fish or fishes)) or stingray* or "sting ray*" or straptail* or "strap tail*" or sturgeon* or (sucker? and (fish or fishes)) or suckerfish* or sunfish* or "sun fish*" or surfclam* or "surf clam*" or surfperch* or surgeonfish* or "surgeon fish*" or sutchi? or swai? or sweetlip* or "sweet lip*" or swordfish* or "sword fish*" or tambaqui* or tang or tangs or tarakihi* or tarpon? or tautog* or tench or tenches or therapon? or thornyhead* or "thorny head*" or threadfin* or "thread fin*" or tigerfish* or "tiger fish*" or tigerperch* or tilapia* or tilefish* or "tile fish*" or (tinfoil* and (fish or fishes)) or toadfish* or "toad fish*" or tomcod* or

		tomtate* or to?ng?esole* or "to?ng?e sole*" or toothfish* or "tooth fish*" or (torpedo* and (fish or fishes)) or tra or tras or trevallies or trevally* or triggerfish* or "trigger fish*" or tripletail* or "tripe tail*" or trout? or trumpeter* or trumpetfish* or "trumpet fish*" or trunkfish* or "trunk fish*" or tullibee* or tuna or tunas or tunicata* or turbot? or turtle? or unicornfish* or "unicorn fish*" or urchin? or vendace* or volute? or wahoo* or walleye* or "wall eye*" or warehou or weakfish* or "weak fish*" or weever* or whale? or whalemeat? or whelk? or whiff? or whiskerfish* or "whisker fish*" or whitefish* or "white fish*" or whiting* or wolffish* or "wolf fish*" or wrasse? or yellowtail* or "yellow tail*" or zander? or zungaro* or "lau lau*" or "mahi mahi*" or "sergeant major*" or "sea cucumber*" or "american smelt*" or "bombay duck*" or "irish lord*" or "pen shell*" or "salmo salar" or "sand lance?" or "spoon worm*" or "striped dwarf*" or "top shell*" or gadiform* or perciform* or decapodiform* or sushi? or sashimi? or ce#iche or se#iche or gravlax or carpaccio or crudo or poke or hinava or "gohu ikan" or esqueixada or kelaguen or namero or kilawin or stroganina or yusheng or "yee sang" or "yuu sahng" or "lo sahng" or koi or kokoda or kuai or lakerda or "larb pla" or "ota ika" or tiradito or xato or umai).ti,ab,kf.
pregnancy	2	exp pregnancy/ or exp pregnancy disorder/ or exp "parameters concerning the fetus, newborn and pregnancy"/ or (pregnan* or prenatal* or "post natal*" or postnatal* or "post natal*" or antenatal* or "ante natal*" or perinatal* or "peri natal*" or postpartum or "post partum" or f?etus* or f?etal or maternal or gravid* or birth*).ti,ab,kf,hw.
lactation	3	lactation/ or exp breast feeding/ or (lactat* or breastfe* or (breast adj (fed or feed*)) or ((human? or breast* or maternal or mother*) adj2 milk*) or breastmilk? or colostrum).ti,ab,kf.
infants	4	exp infant/ or ((("0" or "1" or "2" or zero or one or two) adj5 (age? or year? or old)) or (("0" or "1" or "2" or zero or one or two) adj5 (yr or yrs or y or ys)) or ((month? or mnth? or mth? or ms or m or week? or wks or wk or w or ws or day? or ds or d) adj5 (old or age?)) or infan* or baby or babies or newborn* or "new born*" or neonat* or "neo nat*").ti,ab,kf.
	5	or/2-4
lead	6	lead/ or lead poisoning/ or exp organolead compound/ or lead.hw,kf. or (pb or 208Pb or organolead* or plumbum or plumbic* or plumbate* or plumbous or tetraethyllead or (lead adj8 ("208" or metal* or organometal* or cation* or ion* or isotop* or radioisotop* or element* or microelement* or organic or inorganic or ore or blood* or chemical? or poison* or contam*))).ti,ab,kf.
search structure	7	1 and 5 and 6
not humans	8	(exp animal/ not (exp animal/ and exp human/)) or nonhuman/ or exp animal experiment/ or exp experimental organism/ or (mouse or mice or rat or rats or monkey or monkeys or "preclinical study").ti.
not not humans	9	7 not 8

CENTRAL
Database: CENTRAL
Platform: Cochrane Library
Date of search: October 10, 2024
Limits: Not animal studies

Concept	Line	Search Strategy
seafoods	#1	[mh Fishes] OR [mh "Fish Proteins"] OR [mh ^"Shellfish Proteins"] OR [mh Seafood] OR [mh "Fish Products"] OR [mh Fishes] OR [mh "Fish Proteins"] OR [mh ^"Shellfish Proteins"] OR [mh Seafood] OR [mh "Fish Products"] OR [mh Crustacea] OR [mh Mollusca] OR [mh Cetacea] OR (seafood*:ti,ab,kw OR ("sea" NEXT food*):ti,ab,kw OR ((fish:ti,ab,kw OR fishes:ti,ab,kw OR marine:ti,ab,kw) AND (food*:ti,ab,kw OR diet:ti,ab,kw OR diets:ti,ab,kw OR dieta*:ti,ab,kw OR diete*:ti,ab,kw OR dieti*:ti,ab,kw OR eat*:ti,ab,kw OR ingest*:ti,ab,kw OR nutrit*:ti,ab,kw OR nutrient?:ti,ab,kw OR cuisine*:ti,ab,kw OR culinary:ti,ab,kw OR consum*:ti,ab,kw OR protein?:ti,ab,kw OR product?:ti,ab,kw OR meal:ti,ab,kw)) OR ((fish:ti,ab,kw OR fishes:ti,ab,kw OR marine:ti,ab,kw) NEAR/3 (consum*:ti,ab,kw OR protein?:ti,ab,kw OR product?:ti,ab,kw OR meal:ti,ab,kw OR flour:ti,ab,kw OR fatty:ti,ab,kw)) OR fishmeat?:ti,ab,kw OR abalone*:ti,ab,kw OR aholehole*:ti,ab,kw OR ailia*:ti,ab,kw OR alewife*:ti,ab,kw OR alfonsino*:ti,ab,kw OR amberjack*:ti,ab,kw OR anchov*:ti,ab,kw OR angelfish*:ti,ab,kw OR ("angel" NEXT fish*):ti,ab,kw OR arkshell*:ti,ab,kw OR ("ark" NEXT shell*):ti,ab,kw OR armorhead*:ti,ab,kw OR ("armor" NEXT head*):ti,ab,kw OR arowana*:ti,ab,kw OR ayre*:ti,ab,kw OR bacha?:ti,ab,kw OR bagrid?:ti,ab,kw OR bangamar*:ti,ab,kw OR (barb:ti,ab,kw AND (fish:ti,ab,kw OR fishes:ti,ab,kw)) OR barnacle*:ti,ab,kw OR barracouta*:ti,ab,kw OR barracuda*:ti,ab,kw OR barramundi*:ti,ab,kw OR basa:ti,ab,kw OR bass:ti,ab,kw OR basses:ti,ab,kw OR bata:ti,ab,kw OR beardfish*:ti,ab,kw OR ("beard" NEXT fish*):ti,ab,kw OR bigeye*:ti,ab,kw OR (bittersweet?:ti,ab,kw AND mollusc?:ti,ab,kw) OR blackfish*:ti,ab,kw OR ("black" NEXT fish*):ti,ab,kw OR bluefish*:ti,ab,kw OR ("black" NEXT fish*):ti,ab,kw OR bluegill*:ti,ab,kw OR ("blue" NEXT gill*):ti,ab,kw OR bluenose*:ti,ab,kw OR ("blue" NEXT nose*):ti,ab,kw OR boarfish*:ti,ab,kw OR ("boar" NEXT fish*):ti,ab,kw OR bocachico*:ti,ab,kw OR boga?:ti,ab,kw OR bogue*:ti,ab,kw OR bonefish*:ti,ab,kw OR ("bone" NEXT fish*):ti,ab,kw OR bonito*:ti,ab,kw OR bonnethead*:ti,ab,kw OR ("bonnet" NEXT head*):ti,ab,kw OR bonnetmouth*:ti,ab,kw OR ("bonnet" NEXT mouth*):ti,ab,kw OR bowfin*:ti,ab,kw OR ("bow" NEXT fin*):ti,ab,kw OR bream?:ti,ab,kw OR brotula*:ti,ab,kw OR buffalofish*:ti,ab,kw OR ("buffalo" NEXT fish*):ti,ab,kw OR bullhead?:ti,ab,kw OR ("bull" NEXT head?):ti,ab,kw OR (bumper?:ti,ab,kw AND (fish:ti,ab,kw OR fishes:ti,ab,kw)) OR burbot*:ti,ab,kw OR butterfish*:ti,ab,kw OR (butter* NEXT fish*):ti,ab,kw OR butterflyfish*:ti,ab,kw OR caballa*:ti,ab,kw OR cabrilla*:ti,ab,kw OR cachama*:ti,ab,kw OR caiman*:ti,ab,kw OR calamari*:ti,ab,kw OR calbasu*:ti,ab,kw OR caparari*:ti,ab,kw OR capelin*:ti,ab,kw OR capensis*:ti,ab,kw OR carate*:ti,ab,kw OR cardinalfish*:ti,ab,kw OR ("cardinal" NEXT fish*):ti,ab,kw OR carp:ti,ab,kw OR carplet*:ti,ab,kw OR cascarudo*:ti,ab,kw OR catalina*:ti,ab,kw OR catfish*:ti,ab,kw OR ("cat" NEXT fish*):ti,ab,kw OR catla*:ti,ab,kw OR chanda*:ti,ab,kw OR channa*:ti,ab,kw OR (char?:ti,ab,kw AND (fish:ti,ab,kw OR fishes:ti,ab,kw)) OR (char?:ti,ab,kw NEAR/1

		(arctic:ti,ab,kw OR alsatian:ti,ab,kw OR common:ti,ab,kw OR alpine:ti,ab,kw OR oregon:ti,ab,kw)) OR charal*:ti,ab,kw OR chimaera*:ti,ab,kw OR chiring*:ti,ab,kw OR (chub?:ti,ab,kw AND (fish:ti,ab,kw OR fishes:ti,ab,kw)) OR cichlid?:ti,ab,kw OR cisco*:ti,ab,kw OR clam:ti,ab,kw OR clams:ti,ab,kw OR claresse*:ti,ab,kw OR clarias*:ti,ab,kw OR cobia*:ti,ab,kw OR cockle?:ti,ab,kw OR cod:ti,ab,kw OR conch:ti,ab,kw OR conches:ti,ab,kw OR coquina*:ti,ab,kw OR corbina*:ti,ab,kw OR cornetfish*:ti,ab,kw OR ("cornet" NEXT fish*):ti,ab,kw OR coroata*:ti,ab,kw OR corvina*:ti,ab,kw OR cottonwick*:ti,ab,kw OR ("cotton" NEXT wick*):ti,ab,kw OR cowfish*:ti,ab,kw OR ("cow" NEXT fish*):ti,ab,kw OR cra?fish*:ti,ab,kw OR ("cra?" NEXT fish*):ti,ab,kw OR crab?:ti,ab,kw OR crabmeat*:ti,ab,kw OR crappie*:ti,ab,kw OR crevalle*:ti,ab,kw OR croaker*:ti,ab,kw OR crustacean*:ti,ab,kw OR cubbyu*:ti,ab,kw OR cuirass:ti,ab,kw OR cuirasses:ti,ab,kw OR cunner*:ti,ab,kw OR curimbata*:ti,ab,kw OR cusk?:ti,ab,kw OR cuskeel*:ti,ab,kw OR cutlassfish*:ti,ab,kw OR ("cutlass" NEXT fish*):ti,ab,kw OR cuttlefish*:ti,ab,kw OR ("cuttle" NEXT fish*):ti,ab,kw OR (dab?:ti,ab,kw AND (fish:ti,ab,kw OR fishes:ti,ab,kw)) OR dace:ti,ab,kw OR damselfish*:ti,ab,kw OR ("damsel" NEXT fish*):ti,ab,kw OR dogcockle*:ti,ab,kw OR dorab*:ti,ab,kw OR dories:ti,ab,kw OR dory?:ti,ab,kw OR driftfish*:ti,ab,kw OR ("drift" NEXT fish*):ti,ab,kw OR drum:ti,ab,kw OR drummer*:ti,ab,kw OR duckbill*:ti,ab,kw OR ("duck" NEXT bill*):ti,ab,kw OR eel?:ti,ab,kw OR eelpout*:ti,ab,kw OR em?r?ldperch*:ti,ab,kw OR escargot*:ti,ab,kw OR escolar*:ti,ab,kw OR fanfish*:ti,ab,kw OR ("fan" NEXT fish*):ti,ab,kw OR featherback*:ti,ab,kw OR ("feather" NEXT back*):ti,ab,kw OR fiddler*:ti,ab,kw OR filefish*:ti,ab,kw OR ("file" NEXT fish*):ti,ab,kw OR flathead*:ti,ab,kw OR ("flat" NEXT head*):ti,ab,kw OR flatwhiskered*:ti,ab,kw OR ("flat" NEXT whiskered*):ti,ab,kw OR flounder?:ti,ab,kw OR (fluke?:ti,ab,kw AND (fish:ti,ab,kw OR fishes:ti,ab,kw)) OR flyingfish*:ti,ab,kw OR ("flying" NEXT fish*):ti,ab,kw OR fugu:ti,ab,kw OR fusilier*:ti,ab,kw OR gafftopsail*:ti,ab,kw OR (gag?:ti,ab,kw AND (fish:ti,ab,kw OR fishes:ti,ab,kw)) OR gar:ti,ab,kw OR garfish*:ti,ab,kw OR ("gar" NEXT fish*):ti,ab,kw OR gemfish*:ti,ab,kw OR ("gem" NEXT fish*):ti,ab,kw OR gilleybaka*:ti,ab,kw OR goatfish*:ti,ab,kw OR ("goat" NEXT fish*):ti,ab,kw OR gobies:ti,ab,kw OR goby:ti,ab,kw OR goldeye*:ti,ab,kw OR ("gold" NEXT eye*):ti,ab,kw OR goonch*:ti,ab,kw OR goosefish*:ti,ab,kw OR ("goose" NEXT fish*):ti,ab,kw OR goram*:ti,ab,kw OR gourami*:ti,ab,kw OR grayling*:ti,ab,kw OR greeneye*:ti,ab,kw OR greenling*:ti,ab,kw OR grenadier*:ti,ab,kw OR grouper*:ti,ab,kw OR grunion*:ti,ab,kw OR (grunt?:ti,ab,kw AND (fish:ti,ab,kw OR fishes:ti,ab,kw)) OR grunter*:ti,ab,kw OR guitarfish*:ti,ab,kw OR ("guitar" NEXT fish*):ti,ab,kw OR gurnard*:ti,ab,kw OR haddock*:ti,ab,kw OR hagfish*:ti,ab,kw OR ("hag" NEXT fish*):ti,ab,kw OR hake?:ti,ab,kw OR halfbeaks*:ti,ab,kw OR ("half" NEXT beak*):ti,ab,kw OR halfmoon?:ti,ab,kw OR ("half" NEXT moon*):ti,ab,kw OR halibut*:ti,ab,kw OR hamlet*:ti,ab,kw OR hammerhead*:ti,ab,kw OR ("hammer" NEXT head*):ti,ab,kw OR hassar*:ti,ab,kw OR hatchetfish*:ti,ab,kw OR ("hatchet" NEXT fish*):ti,ab,kw OR hawkfish*:ti,ab,kw OR ("hawk" NEXT fish*):ti,ab,kw OR herring?:ti,ab,kw OR highwaterman*:ti,ab,kw OR hilsa?:ti,ab,kw OR (hind?:ti,ab,kw AND (fish:ti,ab,kw OR fishes:ti,ab,kw)) OR hogfish*:ti,ab,kw OR ("hog" NEXT fish*):ti,ab,kw OR houndfish*:ti,ab,kw OR ("hound" NEXT fish*):ti,ab,kw OR hualca*:ti,ab,kw OR icefish*:ti,ab,kw OR ("ice" NEXT fish*):ti,ab,kw OR inaha*:ti,ab,kw OR isopod*:ti,ab,kw OR jellyfish*:ti,ab,kw OR ("jelly" NEXT fish*):ti,ab,kw OR jewfish*:ti,ab,kw OR ("jew" NEXT fish*):ti,ab,kw OR jobfish*:ti,ab,kw OR ("job" NEXT fish*):ti,ab,kw OR kahawai*:ti,ab,kw OR kajuli*:ti,ab,kw OR kanpachi*:ti,ab,kw OR kelpfish*:ti,ab,kw OR ("kelp" NEXT fish*):ti,ab,kw OR killifish*:ti,ab,kw OR ("killi" NEXT fish*):ti,ab,kw OR kingfish*:ti,ab,kw

		OR ("king" NEXT fish*):ti,ab,kw OR kingklip*:ti,ab,kw OR ("king" NEXT klip*):ti,ab,kw OR knifefish*:ti,ab,kw OR ("knife" NEXT fish*):ti,ab,kw OR knifejaw*:ti,ab,kw OR ("knife" NEXT jaw*):ti,ab,kw OR krill?:ti,ab,kw OR ladyfish*:ti,ab,kw OR ("lady" NEXT fish*):ti,ab,kw OR lancet*:ti,ab,kw OR langostino*:ti,ab,kw OR leaffish*:ti,ab,kw OR ("leaf" NEXT fish*):ti,ab,kw OR leatherjacket*:ti,ab,kw OR ("leather" NEXT jacket*):ti,ab,kw OR limpet*:ti,ab,kw OR (ling?:ti,ab,kw AND (fish:ti,ab,kw OR fishes:ti,ab,kw)) OR lingcod*:ti,ab,kw OR lionfish*:ti,ab,kw OR ("lion" NEXT fish*):ti,ab,kw OR lizardfish*:ti,ab,kw OR ("lizard" NEXT fish*):ti,ab,kw OR loach*:ti,ab,kw OR lobster*:ti,ab,kw OR louvar*:ti,ab,kw OR lumpfish*:ti,ab,kw OR ("lump" NEXT fish*):ti,ab,kw OR lyretail*:ti,ab,kw OR ("lyre" NEXT tail*):ti,ab,kw OR mackerel*:ti,ab,kw OR manta?:ti,ab,kw OR margate*:ti,ab,kw OR marlin?:ti,ab,kw OR megrim*:ti,ab,kw OR menhaden*:ti,ab,kw OR merex*:ti,ab,kw OR miiuy*:ti,ab,kw OR milkfish*:ti,ab,kw OR ("milk" NEXT fish*):ti,ab,kw OR minnow*:ti,ab,kw OR moga?:ti,ab,kw OR mojarra*:ti,ab,kw OR moki?:ti,ab,kw OR mola:ti,ab,kw OR molas:ti,ab,kw OR monkfish*:ti,ab,kw OR ("monk" NEXT fish*):ti,ab,kw OR mooneye*:ti,ab,kw OR ("moon" NEXT eye*):ti,ab,kw OR moonfish*:ti,ab,kw OR ("moon" NEXT fish*):ti,ab,kw OR moonsnail*:ti,ab,kw OR ("moon" NEXT snail*):ti,ab,kw OR morwong*:ti,ab,kw OR mrigal*:ti,ab,kw OR mullet?:ti,ab,kw OR murex:ti,ab,kw OR murexes:ti,ab,kw OR muskellunge*:ti,ab,kw OR mussel?:ti,ab,kw OR mystus:ti,ab,kw OR needlefish*:ti,ab,kw OR ("needle" NEXT fish*):ti,ab,kw OR nodoguro*:ti,ab,kw OR noodlefish*:ti,ab,kw OR ("noodle" NEXT fish*):ti,ab,kw OR octopus*:ti,ab,kw OR octopi:ti,ab,kw OR oilfish*:ti,ab,kw OR ("oil" NEXT fish*):ti,ab,kw OR opah?:ti,ab,kw OR opaleye*:ti,ab,kw OR ("opal" NEXT eye*):ti,ab,kw OR oscar?:ti,ab,kw OR oyster?:ti,ab,kw OR pabdah*:ti,ab,kw OR packoo*:ti,ab,kw OR pacu?:ti,ab,kw OR paddlefish*:ti,ab,kw OR ("paddle" NEXT fish*):ti,ab,kw OR paima?:ti,ab,kw OR pangasius*:ti,ab,kw OR pargo?:ti,ab,kw OR parrotfish*:ti,ab,kw OR ("parrot" NEXT fish*):ti,ab,kw OR pearlspot*:ti,ab,kw OR ("pearl" NEXT spot*):ti,ab,kw OR penshell*:ti,ab,kw OR ("pen" NEXT shell*):ti,ab,kw OR perch:ti,ab,kw OR perches:ti,ab,kw OR periwinkle?:ti,ab,kw OR picarel*:ti,ab,kw OR pickerel*:ti,ab,kw OR pike?:ti,ab,kw OR pikeperch*:ti,ab,kw OR pilchard*:ti,ab,kw OR pintado*:ti,ab,kw OR pipefish*:ti,ab,kw OR ("pipe" NEXT fish*):ti,ab,kw OR piramutaba*:ti,ab,kw OR pirarucu*:ti,ab,kw OR plaice*:ti,ab,kw OR pleco?:ti,ab,kw OR pollock?:ti,ab,kw OR pomfret?:ti,ab,kw OR pompanito*:ti,ab,kw OR pompano*:ti,ab,kw OR ponyfish*:ti,ab,kw OR ("pony" NEXT fish*):ti,ab,kw OR porgy?:ti,ab,kw OR porgies:ti,ab,kw OR porkfish*:ti,ab,kw OR ("pork" NEXT fish*):ti,ab,kw OR potasi?:ti,ab,kw OR pout?:ti,ab,kw OR prawn?:ti,ab,kw OR puffer*:ti,ab,kw OR punti?:ti,ab,kw OR quahog*:ti,ab,kw OR queenfish*:ti,ab,kw OR ("queen" NEXT fish*):ti,ab,kw OR (racehorse?:ti,ab,kw AND (fish:ti,ab,kw OR fishes:ti,ab,kw)) OR ((ray:ti,ab,kw OR rays:ti,ab,kw) AND (fish:ti,ab,kw OR fishes:ti,ab,kw)) OR redfish*:ti,ab,kw OR ("red" NEXT fish*):ti,ab,kw OR redhorse*:ti,ab,kw OR ("red" NEXT horse*):ti,ab,kw OR remora?:ti,ab,kw OR rita?:ti,ab,kw OR rockfish*:ti,ab,kw OR ("rock" NEXT fish*):ti,ab,kw OR rockling*:ti,ab,kw OR rohu?:ti,ab,kw OR roosterfish*:ti,ab,kw OR ("rooster" NEXT fish*):ti,ab,kw OR rosefish*:ti,ab,kw OR ("rose" NEXT fish*):ti,ab,kw OR roughback*:ti,ab,kw OR ("rough" NEXT back*):ti,ab,kw OR roughies:ti,ab,kw OR roughy*:ti,ab,kw OR ruff:ti,ab,kw OR (runner?:ti,ab,kw AND (fish:ti,ab,kw OR fishes:ti,ab,kw)) OR sablefish*:ti,ab,kw OR ("sable" NEXT fish*):ti,ab,kw OR sailfish*:ti,ab,kw OR ("sail" NEXT fish*):ti,ab,kw OR salmon*:ti,ab,kw OR sampa?:ti,ab,kw OR sanddab*:ti,ab,kw OR sandeel*:ti,ab,kw OR (sander?:ti,ab,kw AND (fish:ti,ab,kw OR fishes:ti,ab,kw)) OR sandfish*:ti,ab,kw OR ("sand" NEXT fish*):ti,ab,kw OR

		sandperch*:ti,ab,kw OR sardine?:ti,ab,kw OR sargo?:ti,ab,kw OR sauger?:ti,ab,kw OR sauries:ti,ab,kw OR saury*:ti,ab,kw OR sawfish*:ti,ab,kw OR ("saw" NEXT fish*):ti,ab,kw OR scad?:ti,ab,kw OR scallop?:ti,ab,kw OR scamp?:ti,ab,kw OR schoolmaster*:ti,ab,kw OR scorpionfish*:ti,ab,kw OR ("scorpion" NEXT fish*):ti,ab,kw OR scup:ti,ab,kw OR scups:ti,ab,kw OR scupper?:ti,ab,kw OR seabass*:ti,ab,kw OR seabob*:ti,ab,kw OR ("sea" NEXT bob*):ti,ab,kw OR seabream*:ti,ab,kw OR searobin*:ti,ab,kw OR ("sea" NEXT robin*):ti,ab,kw OR seasnail*:ti,ab,kw OR ("sea" NEXT snail*):ti,ab,kw OR shad:ti,ab,kw OR shads:ti,ab,kw OR shadefish*:ti,ab,kw OR ("shade" NEXT fish*):ti,ab,kw OR shark?:ti,ab,kw OR sharksucker?:ti,ab,kw OR sheatfish*:ti,ab,kw OR ("sheat" NEXT fish*):ti,ab,kw OR sheep?head?:ti,ab,kw OR (sheep? NEXT head?):ti,ab,kw OR shellfish*:ti,ab,kw OR ("shell" NEXT fish*):ti,ab,kw OR shiner?:ti,ab,kw OR shrimp?:ti,ab,kw OR sild:ti,ab,kw OR silds:ti,ab,kw OR sillago*:ti,ab,kw OR silverside*:ti,ab,kw OR ("silver" NEXT side*):ti,ab,kw OR skate?:ti,ab,kw OR skilfish*:ti,ab,kw OR ("skil" NEXT fish*):ti,ab,kw OR slipmouth*:ti,ab,kw OR ("slip" NEXT mouth*):ti,ab,kw OR slipperysnail*:ti,ab,kw OR ("slippery" NEXT snail*):ti,ab,kw OR (smelt*:ti,ab,kw AND (fish:ti,ab,kw OR fishes:ti,ab,kw)) OR smoothhound*:ti,ab,kw OR ("smooth" NEXT hound*):ti,ab,kw OR snail?:ti,ab,kw OR snakehead?:ti,ab,kw OR snapper?:ti,ab,kw OR snook?:ti,ab,kw OR soldierfish*:ti,ab,kw OR ("soldier" NEXT fish*):ti,ab,kw OR (sole*:ti,ab,kw AND (fish:ti,ab,kw OR fishes:ti,ab,kw)) OR s?rubi?:ti,ab,kw OR spadefish*:ti,ab,kw OR ("spade" NEXT fish*):ti,ab,kw OR spearfish*:ti,ab,kw OR ("spear" NEXT fish*):ti,ab,kw OR spinef??t*:ti,ab,kw OR ("spine" NEXT f??t*):ti,ab,kw OR (spot*:ti,ab,kw AND (fish:ti,ab,kw OR fishes:ti,ab,kw)) OR sprat:ti,ab,kw OR sprats:ti,ab,kw OR squid?:ti,ab,kw OR squirrelfish*:ti,ab,kw OR ("squirrel" NEXT fish*):ti,ab,kw OR stargazer*:ti,ab,kw OR ("star" NEXT gazer*):ti,ab,kw OR (stinging:ti,ab,kw AND (fish:ti,ab,kw OR fishes:ti,ab,kw)) OR stingray*:ti,ab,kw OR ("sting" NEXT ray*):ti,ab,kw OR straptail*:ti,ab,kw OR ("strap" NEXT tail*):ti,ab,kw OR sturgeon*:ti,ab,kw OR (sucker?:ti,ab,kw AND (fish:ti,ab,kw OR fishes:ti,ab,kw)) OR suckerfish*:ti,ab,kw OR sunfish*:ti,ab,kw OR ("sun" NEXT fish*):ti,ab,kw OR surfclam*:ti,ab,kw OR ("surf" NEXT clam*):ti,ab,kw OR surfperch*:ti,ab,kw OR surgeonfish*:ti,ab,kw OR ("surgeon" NEXT fish*):ti,ab,kw OR sutchi?:ti,ab,kw OR swai?:ti,ab,kw OR sweetlip*:ti,ab,kw OR ("sweet" NEXT lip*):ti,ab,kw OR swordfish*:ti,ab,kw OR ("sword" NEXT fish*):ti,ab,kw OR tambaqui*:ti,ab,kw OR tang:ti,ab,kw OR tangs:ti,ab,kw OR tarakihi*:ti,ab,kw OR tarpon?:ti,ab,kw OR tautog*:ti,ab,kw OR tench:ti,ab,kw OR tenches:ti,ab,kw OR therapon?:ti,ab,kw OR thornyhead*:ti,ab,kw OR ("thorny" NEXT head*):ti,ab,kw OR threadfin*:ti,ab,kw OR ("thread" NEXT fin*):ti,ab,kw OR tigerfish*:ti,ab,kw OR ("tiger" NEXT fish*):ti,ab,kw OR tigerperch*:ti,ab,kw OR tilapia*:ti,ab,kw OR tilefish*:ti,ab,kw OR ("tile" NEXT fish*):ti,ab,kw OR (tinfoil*:ti,ab,kw AND (fish:ti,ab,kw OR fishes:ti,ab,kw)) OR toadfish*:ti,ab,kw OR ("toad" NEXT fish*):ti,ab,kw OR tomcod*:ti,ab,kw OR tomtate*:ti,ab,kw OR to?ng?esole*:ti,ab,kw OR (to?ng?e NEXT sole*):ti,ab,kw OR toothfish*:ti,ab,kw OR ("tooth" NEXT fish*):ti,ab,kw OR (torpedo*:ti,ab,kw AND (fish:ti,ab,kw OR fishes:ti,ab,kw)) OR tra:ti,ab,kw OR tras:ti,ab,kw OR trevallies:ti,ab,kw OR trevally*:ti,ab,kw OR triggerfish*:ti,ab,kw OR ("trigger" NEXT fish*):ti,ab,kw OR tripletail*:ti,ab,kw OR ("tripe" NEXT tail*):ti,ab,kw OR trout?:ti,ab,kw OR trumpeter*:ti,ab,kw OR trumpetfish*:ti,ab,kw OR ("trumpet" NEXT fish*):ti,ab,kw OR trunkfish*:ti,ab,kw OR ("trunk" NEXT fish*):ti,ab,kw OR tullibee*:ti,ab,kw OR tuna:ti,ab,kw OR tunas:ti,ab,kw OR tunicata*:ti,ab,kw OR turbot?:ti,ab,kw OR turtle?:ti,ab,kw OR unicornfish*:ti,ab,kw OR ("unicorn" NEXT fish*):ti,ab,kw OR urchin?:ti,ab,kw OR

		vendace*:ti,ab,kw OR volute?:ti,ab,kw OR wahoo*:ti,ab,kw OR walleye*:ti,ab,kw OR ("wall" NEXT eye*):ti,ab,kw OR warehou:ti,ab,kw OR weakfish*:ti,ab,kw OR ("weak" NEXT fish*):ti,ab,kw OR weever*:ti,ab,kw OR whale?:ti,ab,kw OR whalemeat?:ti,ab,kw OR whelk?:ti,ab,kw OR whiff?:ti,ab,kw OR whiskerfish*:ti,ab,kw OR ("whisker" NEXT fish*):ti,ab,kw OR whitefish*:ti,ab,kw OR ("white" NEXT fish*):ti,ab,kw OR whiting*:ti,ab,kw OR wolffish*:ti,ab,kw OR ("wolf" NEXT fish*):ti,ab,kw OR wrasse?:ti,ab,kw OR yellowtail*:ti,ab,kw OR ("yellow" NEXT tail*):ti,ab,kw OR zander?:ti,ab,kw OR zungaro*:ti,ab,kw OR ("lau" NEXT lau*):ti,ab,kw OR ("mahi" NEXT mahi*):ti,ab,kw OR ("sergeant" NEXT major*):ti,ab,kw OR ("sea" NEXT cucumber*):ti,ab,kw OR ("american" NEXT smelt*):ti,ab,kw OR ("bombay" NEXT duck*):ti,ab,kw OR ("irish" NEXT lord*):ti,ab,kw OR ("pen" NEXT shell*):ti,ab,kw OR "salmo salar":ti,ab,kw OR ("sand" NEXT lance?):ti,ab,kw OR ("spoon" NEXT worm*):ti,ab,kw OR ("striped" NEXT dwarf*):ti,ab,kw OR ("top" NEXT shell*):ti,ab,kw OR gadiform*:ti,ab,kw OR perciform*:ti,ab,kw OR decapodiform*:ti,ab,kw OR sushi?:ti,ab,kw OR sashimi?:ti,ab,kw OR ce?iche:ti,ab,kw OR se?iche:ti,ab,kw OR gravlax:ti,ab,kw OR carpaccio:ti,ab,kw OR crudo:ti,ab,kw OR poke:ti,ab,kw OR hinava:ti,ab,kw OR "gohu ikan":ti,ab,kw OR esqueixada:ti,ab,kw OR kelaguen:ti,ab,kw OR namero:ti,ab,kw OR kilawin:ti,ab,kw OR stroganina:ti,ab,kw OR yusheng:ti,ab,kw OR "yee sang":ti,ab,kw OR "yuu sahng":ti,ab,kw OR "lo sahng":ti,ab,kw OR koi:ti,ab,kw OR kokoda:ti,ab,kw OR kuai:ti,ab,kw OR lakerda:ti,ab,kw OR "larb pla":ti,ab,kw OR "ota ika":ti,ab,kw OR tiradito:ti,ab,kw OR xato:ti,ab,kw OR umai:ti,ab,kw)
pregnancy	#2	[mh Pregnancy] OR [mh "Pregnancy Complications"] OR [mh "Pregnancy Outcome"] OR [mh Fetus] OR [mh ^"Pregnant Women"] OR [mh ^"Maternal Exposure"] OR (pregnan*:ti,ab,kw OR prenatal*:ti,ab,kw OR ("post" NEXT natal*):ti,ab,kw OR postnatal*:ti,ab,kw OR ("post" NEXT natal*):ti,ab,kw OR antenatal*:ti,ab,kw OR ("ante" NEXT natal*):ti,ab,kw OR perinatal*:ti,ab,kw OR ("peri" NEXT natal*):ti,ab,kw OR postpartum:ti,ab,kw OR "post partum":ti,ab,kw OR f?etus*:ti,ab,kw OR f?etal:ti,ab,kw OR maternal:ti,ab,kw OR gravid*:ti,ab,kw OR birth*:ti,ab,kw)
lactation	#3	[mh Lactation] OR [mh "Breast Feeding"] OR [mh "Milk, Human"] OR (lactat*:ti,ab,kw OR breastfe*:ti,ab,kw OR (breast:ti,ab,kw NEXT (fed:ti,ab,kw OR feed*:ti,ab,kw)) OR ((human?:ti,ab,kw OR breast*:ti,ab,kw OR maternal:ti,ab,kw OR mother*:ti,ab,kw) NEAR/2 milk*:ti,ab,kw) OR breastmilk?:ti,ab,kw OR colostrum:ti,ab,kw)
infants	#4	[mh Infant] OR (((0:ti,ab,kw OR 1:ti,ab,kw OR 2:ti,ab,kw OR zero:ti,ab,kw OR one:ti,ab,kw OR two:ti,ab,kw) NEAR/5 (age?:ti,ab,kw OR year?:ti,ab,kw OR old:ti,ab,kw)) OR ((0:ti,ab,kw OR 1:ti,ab,kw OR 2:ti,ab,kw OR zero:ti,ab,kw OR one:ti,ab,kw OR two:ti,ab,kw) NEAR/5 (yr:ti,ab,kw OR yrs:ti,ab,kw OR y:ti,ab,kw OR ys:ti,ab,kw)) OR ((month?:ti,ab,kw OR mnth?:ti,ab,kw OR mth?:ti,ab,kw OR ms:ti,ab,kw OR m:ti,ab,kw OR week?:ti,ab,kw OR wks:ti,ab,kw OR wk:ti,ab,kw OR "w":ti,ab,kw OR ws:ti,ab,kw OR day?:ti,ab,kw OR ds:ti,ab,kw OR d:ti,ab,kw) NEAR/5 (old:ti,ab,kw OR age?:ti,ab,kw)) OR infan*:ti,ab,kw OR baby:ti,ab,kw OR babies:ti,ab,kw OR newborn*:ti,ab,kw OR ("new" NEXT born*):ti,ab,kw OR neonat*:ti,ab,kw OR ("neo" NEXT nat*):ti,ab,kw)
	#5	#2 OR #3 OR #4
lead	#6	[mh "Lead Poisoning"] OR [mh Lead] OR [mh "Lead Radioisotopes"] OR [mh "Tetraethyl Lead"] OR lead:kw OR (pb:ti,ab,kw OR 208Pb:ti,ab,kw OR organolead*:ti,ab,kw OR plumbum:ti,ab,kw OR plumbic*:ti,ab,kw OR plumbate*:ti,ab,kw OR plumbous:ti,ab,kw OR tetraethyllead:ti,ab,kw OR (lead:ti,ab,kw NEAR/8 (208:ti,ab,kw OR metal*:ti,ab,kw OR organometal*:ti,ab,kw OR cation*:ti,ab,kw OR ion*:ti,ab,kw OR isotop*:ti,ab,kw OR radioisotop*:ti,ab,kw OR element*:ti,ab,kw OR microelement*:ti,ab,kw OR organic:ti,ab,kw

		OR inorganic:ti,ab,kw OR ore:ti,ab,kw OR blood*:ti,ab,kw OR chemical?:ti,ab,kw OR poison*:ti,ab,kw OR contam*:ti,ab,kw)))
search structure	#7	#1 AND #5 AND #6
not humans	#8	([mh Animals] NOT ([mh Animals] AND [mh ^Humans])) OR [mh "Animal Experimentation"] OR [mh "Models, Animal"] OR (mouse:ti OR mice:ti OR rat:ti OR rats:ti OR monkey:ti OR monkeys:ti OR "preclinical study":ti)
not not humans	#9	#7 NOT #8 in Trials

APA PsycInfo
Database: APA PsycInfo
Platform: EBSCO
Date of search: October 10, 2024
Limits: Not animal studies
Expanders: Apply related words, Apply equivalent subjects

Concept	Line	Search Strategy
seafoods	S1	(DE "Fishes" OR DE "Bass (Fish)" OR DE "Carp" OR DE "Cichlids" OR DE "Electric Fishes" OR DE "Salmon" OR DE "Sticklebacks" OR DE "Goldfish" OR DE "Crustacea" OR DE "Crabs" OR DE "Crayfish" OR DE "Whales" OR DE "Dolphins" OR DE "Porpoises") OR ((TI seafood* OR AB seafood* OR SU seafood*) OR (TI "sea food*" OR AB "sea food*" OR SU "sea food*") OR (((TI fish OR AB fish OR SU fish) OR (TI fishes OR AB fishes OR SU fishes) OR (TI marine OR AB marine OR SU marine)) AND ((TI food* OR AB food* OR SU food*) OR (TI diet OR AB diet OR SU diet) OR (TI diets OR AB diets OR SU diets) OR (TI dieta* OR AB dieta* OR SU dieta*) OR (TI diete* OR AB diete* OR SU diete*) OR (TI dieti* OR AB dieti* OR SU dieti*) OR (TI eat* OR AB eat* OR SU eat*) OR (TI ingest* OR AB ingest* OR SU ingest*) OR (TI nutrit* OR AB nutrit* OR SU nutrit*) OR (TI nutrient# OR AB nutrient# OR SU nutrient#) OR (TI cuisine* OR AB cuisine* OR SU cuisine*) OR (TI culinary OR AB culinary OR SU culinary) OR (TI consum* OR AB consum* OR SU consum*) OR (TI protein# OR AB protein# OR SU protein#) OR (TI product# OR AB product# OR SU product#) OR (TI meal OR AB meal OR SU meal))) OR (((TI fish OR AB fish OR SU fish) OR (TI fishes OR AB fishes OR SU fishes) OR (TI marine OR AB marine OR SU marine)) N3 ((TI consum* OR AB consum* OR SU consum*) OR (TI protein# OR AB protein# OR SU protein#) OR (TI product# OR AB product# OR SU product#) OR (TI meal OR AB meal OR SU meal) OR (TI flour OR AB flour OR SU flour) OR (TI fatty OR AB fatty OR SU fatty))) OR (TI fishmeat# OR AB fishmeat# OR SU fishmeat#) OR (TI abalone* OR AB abalone* OR SU abalone*) OR (TI aholehole* OR AB aholehole* OR SU aholehole*) OR (TI ailia* OR AB ailia* OR SU ailia*) OR (TI alewife* OR AB alewife* OR SU alewife*) OR (TI alfonsino* OR AB alfonsino* OR SU alfonsino*) OR (TI amberjack* OR AB amberjack* OR SU amberjack*) OR (TI anchov* OR AB anchov* OR SU anchov*) OR (TI angelfish* OR AB angelfish* OR SU angelfish*) OR (TI "angel fish*" OR AB "angel fish*" OR SU "angel fish*") OR (TI arkshell* OR AB arkshell* OR SU arkshell*) OR (TI "ark shell*" OR AB "ark shell*" OR SU "ark shell*") OR (TI armorhead* OR AB armorhead* OR SU armorhead*) OR (TI "armor head*" OR AB "armor head*" OR SU "armor head*") OR (TI arowana* OR AB arowana* OR SU arowana*) OR (TI ayre* OR AB ayre* OR SU ayre*) OR (TI bacha# OR AB bacha# OR SU bacha#) OR (TI bagrid# OR AB bagrid# OR SU bagrid#) OR (TI bangamar* OR AB bangamar* OR SU bangamar*) OR ((TI barb OR AB barb OR SU barb) AND ((TI fish OR AB fish OR SU fish) OR (TI fishes OR AB fishes OR SU fishes))) OR (TI barnacle* OR AB barnacle* OR SU barnacle*) OR (TI barracouta* OR AB barracouta* OR SU barracouta*) OR (TI barracuda* OR AB barracuda* OR SU barracuda*) OR (TI barramundi* OR AB barramundi* OR SU barramundi*) OR (TI basa OR AB basa OR SU basa) OR (TI bass OR AB bass OR SU bass) OR (TI basses OR AB basses OR SU basses) OR (TI bata OR AB bata OR SU bata) OR (TI beardfish* OR AB beardfish* OR SU beardfish*) OR (TI "beard fish*" OR AB "beard fish*" OR SU "beard fish*") OR (TI bigeye* OR AB bigeye* OR SU bigeye*) OR ((TI bittersweet# OR AB bittersweet# OR SU bittersweet#) AND (TI mollusc# OR AB mollusc# OR SU mollusc#)) OR (TI blackfish* OR AB blackfish* OR SU blackfish*) OR (TI

		"black fish*" OR AB "black fish*" OR SU "black fish*") OR (TI bluefish* OR AB bluefish* OR SU bluefish*) OR (TI "black fish*" OR AB "black fish*" OR SU "black fish*") OR (TI bluegill* OR AB bluegill* OR SU bluegill*) OR (TI "blue gill*" OR AB "blue gill*" OR SU "blue gill*") OR (TI bluenose* OR AB bluenose* OR SU bluenose*) OR (TI "blue nose*" OR AB "blue nose*" OR SU "blue nose*") OR (TI boarfish* OR AB boarfish* OR SU boarfish*) OR (TI "boar fish*" OR AB "boar fish*" OR SU "boar fish*") OR (TI bocachico* OR AB bocachico* OR SU bocachico*) OR (TI boga# OR AB boga# OR SU boga#) OR (TI bogue* OR AB bogue* OR SU bogue*) OR (TI bonefish* OR AB bonefish* OR SU bonefish*) OR (TI "bone fish*" OR AB "bone fish*" OR SU "bone fish*") OR (TI bonito* OR AB bonito* OR SU bonito*) OR (TI bonnethead* OR AB bonnethead* OR SU bonnethead*) OR (TI "bonnet head*" OR AB "bonnet head*" OR SU "bonnet head*") OR (TI bonnetmouth* OR AB bonnetmouth* OR SU bonnetmouth*) OR (TI "bonnet mouth*" OR AB "bonnet mouth*" OR SU "bonnet mouth*") OR (TI bowfin* OR AB bowfin* OR SU bowfin*) OR (TI "bow fin*" OR AB "bow fin*" OR SU "bow fin*") OR (TI bream# OR AB bream# OR SU bream#) OR (TI brotula* OR AB brotula* OR SU brotula*) OR (TI buffalofish* OR AB buffalofish* OR SU buffalofish*) OR (TI "buffalo fish*" OR AB "buffalo fish*" OR SU "buffalo fish*") OR (TI bullhead# OR AB bullhead# OR SU bullhead#) OR (TI "bull head#" OR AB "bull head#" OR SU "bull head#") OR ((TI bumper# OR AB bumper# OR SU bumper#) AND ((TI fish OR AB fish OR SU fish) OR (TI fishes OR AB fishes OR SU fishes))) OR (TI burbot* OR AB burbot* OR SU burbot*) OR (TI butterfish* OR AB butterfish* OR SU butterfish*) OR (TI "butter* fish*" OR AB "butter* fish*" OR SU "butter* fish*") OR (TI butterflyfish* OR AB butterflyfish* OR SU butterflyfish*) OR (TI caballa* OR AB caballa* OR SU caballa*) OR (TI cabrilla* OR AB cabrilla* OR SU cabrilla*) OR (TI cachama* OR AB cachama* OR SU cachama*) OR (TI caiman* OR AB caiman* OR SU caiman*) OR (TI calamari* OR AB calamari* OR SU calamari*) OR (TI calbasu* OR AB calbasu* OR SU calbasu*) OR (TI caparari* OR AB caparari* OR SU caparari*) OR (TI capelin* OR AB capelin* OR SU capelin*) OR (TI capensis* OR AB capensis* OR SU capensis*) OR (TI carate* OR AB carate* OR SU carate*) OR (TI cardinalfish* OR AB cardinalfish* OR SU cardinalfish*) OR (TI "cardinal fish*" OR AB "cardinal fish*" OR SU "cardinal fish*") OR (TI carp OR AB carp OR SU carp) OR (TI carplet* OR AB carplet* OR SU carplet*) OR (TI cascarudo* OR AB cascarudo* OR SU cascarudo*) OR (TI catalina* OR AB catalina* OR SU catalina*) OR (TI catfish* OR AB catfish* OR SU catfish*) OR (TI "cat fish*" OR AB "cat fish*" OR SU "cat fish*") OR (TI catla* OR AB catla* OR SU catla*) OR (TI chanda* OR AB chanda* OR SU chanda*) OR (TI channa* OR AB channa* OR SU channa*) OR ((TI char# OR AB char# OR SU char#) AND ((TI fish OR AB fish OR SU fish) OR (TI fishes OR AB fishes OR SU fishes))) OR ((TI char# OR AB char# OR SU char#) N1 ((TI arctic OR AB arctic OR SU arctic) OR (TI alsatian OR AB alsatian OR SU alsatian) OR (TI common OR AB common OR SU common) OR (TI alpine OR AB alpine OR SU alpine) OR (TI oregon OR AB oregon OR SU oregon))) OR (TI charal* OR AB charal* OR SU charal*) OR (TI chimaera* OR AB chimaera* OR SU chimaera*) OR (TI chiring* OR AB chiring* OR SU chiring*) OR ((TI chub# OR AB chub# OR SU chub#) AND ((TI fish OR AB fish OR SU fish) OR (TI fishes OR AB fishes OR SU fishes))) OR (TI cichlid# OR AB cichlid# OR SU cichlid#) OR (TI cisco* OR AB cisco* OR SU cisco*) OR (TI clam OR AB clam OR SU clam) OR (TI clams OR AB clams OR SU clams) OR (TI claresse* OR AB claresse* OR SU claresse*) OR (TI clarias* OR AB clarias* OR SU clarias*) OR (TI cobia* OR AB cobia* OR SU cobia*) OR (TI cockle# OR AB cockle# OR SU cockle#) OR (TI cod OR AB cod OR SU cod) OR (TI conch OR AB conch OR SU conch) OR (TI conches OR AB conches OR SU conches) OR (TI coquina* OR AB coquina* OR SU coquina*) OR (TI corbina* OR AB corbina* OR SU corbina*) OR (TI

		cornetfish* OR AB cornetfish* OR SU cornetfish*) OR (TI "cornet fish*" OR AB "cornet fish*" OR SU "cornet fish*") OR (TI coroata* OR AB coroata* OR SU coroata*) OR (TI corvina* OR AB corvina* OR SU corvina*) OR (TI cottonwick* OR AB cottonwick* OR SU cottonwick*) OR (TI "cotton wick*" OR AB "cotton wick*" OR SU "cotton wick*") OR (TI cowfish* OR AB cowfish* OR SU cowfish*) OR (TI "cow fish*" OR AB "cow fish*" OR SU "cow fish*") OR (TI cra?fish* OR AB cra?fish* OR SU cra?fish*) OR (TI "cra? fish*" OR AB "cra? fish*" OR SU "cra? fish*") OR (TI crab# OR AB crab# OR SU crab#) OR (TI crabmeat* OR AB crabmeat* OR SU crabmeat*) OR (TI crappie* OR AB crappie* OR SU crappie*) OR (TI crevalle* OR AB crevalle* OR SU crevalle*) OR (TI croaker* OR AB croaker* OR SU croaker*) OR (TI crustacean* OR AB crustacean* OR SU crustacean*) OR (TI cubbyu* OR AB cubbyu* OR SU cubbyu*) OR (TI cuirass OR AB cuirass OR SU cuirass) OR (TI cuirasses OR AB cuirasses OR SU cuirasses) OR (TI cunner* OR AB cunner* OR SU cunner*) OR (TI curimbata* OR AB curimbata* OR SU curimbata*) OR (TI cusk# OR AB cusk# OR SU cusk#) OR (TI cuskeel* OR AB cuskeel* OR SU cuskeel*) OR (TI cutlassfish* OR AB cutlassfish* OR SU cutlassfish*) OR (TI "cutlass fish*" OR AB "cutlass fish*" OR SU "cutlass fish*") OR (TI cuttlefish* OR AB cuttlefish* OR SU cuttlefish*) OR (TI "cuttle fish*" OR AB "cuttle fish*" OR SU "cuttle fish*") OR ((TI dab# OR AB dab# OR SU dab#) AND ((TI fish OR AB fish OR SU fish) OR (TI fishes OR AB fishes OR SU fishes))) OR (TI dace OR AB dace OR SU dace) OR (TI damselfish* OR AB damselfish* OR SU damselfish*) OR (TI "damsel fish*" OR AB "damsel fish*" OR SU "damsel fish*") OR (TI dogcockle* OR AB dogcockle* OR SU dogcockle*) OR (TI dorab* OR AB dorab* OR SU dorab*) OR (TI dories OR AB dories OR SU dories) OR (TI dory# OR AB dory# OR SU dory#) OR (TI driftfish* OR AB driftfish* OR SU driftfish*) OR (TI "drift fish*" OR AB "drift fish*" OR SU "drift fish*") OR (TI drum OR AB drum OR SU drum) OR (TI drummer* OR AB drummer* OR SU drummer*) OR (TI duckbill* OR AB duckbill* OR SU duckbill*) OR (TI "duck bill*" OR AB "duck bill*" OR SU "duck bill*") OR (TI eel# OR AB eel# OR SU eel#) OR (TI eelpout* OR AB eelpout* OR SU eelpout*) OR (TI em#r?ldperch* OR AB em#r?ldperch* OR SU em#r?ldperch*) OR (TI escargot* OR AB escargot* OR SU escargot*) OR (TI escolar* OR AB escolar* OR SU escolar*) OR (TI fanfish* OR AB fanfish* OR SU fanfish*) OR (TI "fan fish*" OR AB "fan fish*" OR SU "fan fish*") OR (TI featherback* OR AB featherback* OR SU featherback*) OR (TI "feather back*" OR AB "feather back*" OR SU "feather back*") OR (TI fiddler* OR AB fiddler* OR SU fiddler*) OR (TI filefish* OR AB filefish* OR SU filefish*) OR (TI "file fish*" OR AB "file fish*" OR SU "file fish*") OR (TI flathead* OR AB flathead* OR SU flathead*) OR (TI "flat head*" OR AB "flat head*" OR SU "flat head*") OR (TI flatwhiskered* OR AB flatwhiskered* OR SU flatwhiskered*) OR (TI "flat whiskered*" OR AB "flat whiskered*" OR SU "flat whiskered*") OR (TI flounder# OR AB flounder# OR SU flounder#) OR ((TI fluke# OR AB fluke# OR SU fluke#) AND ((TI fish OR AB fish OR SU fish) OR (TI fishes OR AB fishes OR SU fishes))) OR (TI flyingfish* OR AB flyingfish* OR SU flyingfish*) OR (TI "flying fish*" OR AB "flying fish*" OR SU "flying fish*") OR (TI fugu OR AB fugu OR SU fugu) OR (TI fusilier* OR AB fusilier* OR SU fusilier*) OR (TI gafftopsail* OR AB gafftopsail* OR SU gafftopsail*) OR ((TI gag# OR AB gag# OR SU gag#) AND ((TI fish OR AB fish OR SU fish) OR (TI fishes OR AB fishes OR SU fishes))) OR (TI gar OR AB gar OR SU gar) OR (TI garfish* OR AB garfish* OR SU garfish*) OR (TI "gar fish*" OR AB "gar fish*" OR SU "gar fish*") OR (TI gemfish* OR AB gemfish* OR SU gemfish*) OR (TI "gem fish*" OR AB "gem fish*" OR SU "gem fish*") OR (TI gilleybaka* OR AB gilleybaka* OR SU gilleybaka*) OR (TI goatfish* OR AB goatfish* OR SU goatfish*) OR (TI "goat fish*" OR AB "goat fish*" OR SU "goat fish*") OR (TI gobies OR AB gobies OR SU gobies) OR (TI goby OR AB goby OR SU goby) OR (TI goldeye* OR AB goldeye* OR SU goldeye*) OR (TI "gold

		eye*" OR AB "gold eye*" OR SU "gold eye*") OR (TI goonch* OR AB goonch* OR SU goonch*) OR (TI goosefish* OR AB goosefish* OR SU goosefish*) OR (TI "goose fish*" OR AB "goose fish*" OR SU "goose fish*") OR (TI goram* OR AB goram* OR SU goram*) OR (TI gourami* OR AB gourami* OR SU gourami*) OR (TI grayling* OR AB grayling* OR SU grayling*) OR (TI greeneye* OR AB greeneye* OR SU greeneye*) OR (TI greenling* OR AB greenling* OR SU greenling*) OR (TI grenadier* OR AB grenadier* OR SU grenadier*) OR (TI grouper* OR AB grouper* OR SU grouper*) OR (TI grunion* OR AB grunion* OR SU grunion*) OR ((TI grunt# OR AB grunt# OR SU grunt#) AND ((TI fish OR AB fish OR SU fish) OR (TI fishes OR AB fishes OR SU fishes))) OR (TI grunter* OR AB grunter* OR SU grunter*) OR (TI guitarfish* OR AB guitarfish* OR SU guitarfish*) OR (TI "guitar fish*" OR AB "guitar fish*" OR SU "guitar fish*") OR (TI gurnard* OR AB gurnard* OR SU gurnard*) OR (TI haddock* OR AB haddock* OR SU haddock*) OR (TI hagfish* OR AB hagfish* OR SU hagfish*) OR (TI "hag fish*" OR AB "hag fish*" OR SU "hag fish*") OR (TI hake# OR AB hake# OR SU hake#) OR (TI halfbeaks* OR AB halfbeaks* OR SU halfbeaks*) OR (TI "half beak*" OR AB "half beak*" OR SU "half beak*") OR (TI halfmoon# OR AB halfmoon# OR SU halfmoon#) OR (TI "half moon*" OR AB "half moon*" OR SU "half moon*") OR (TI halibut* OR AB halibut* OR SU halibut*) OR (TI hamlet* OR AB hamlet* OR SU hamlet*) OR (TI hammerhead* OR AB hammerhead* OR SU hammerhead*) OR (TI "hammer head*" OR AB "hammer head*" OR SU "hammer head*") OR (TI hassar* OR AB hassar* OR SU hassar*) OR (TI hatchetfish* OR AB hatchetfish* OR SU hatchetfish*) OR (TI "hatchet fish*" OR AB "hatchet fish*" OR SU "hatchet fish*") OR (TI hawkfish* OR AB hawkfish* OR SU hawkfish*) OR (TI "hawk fish*" OR AB "hawk fish*" OR SU "hawk fish*") OR (TI herring# OR AB herring# OR SU herring#) OR (TI highwaterman* OR AB highwaterman* OR SU highwaterman*) OR (TI hilsa# OR AB hilsa# OR SU hilsa#) OR ((TI hind# OR AB hind# OR SU hind#) AND ((TI fish OR AB fish OR SU fish) OR (TI fishes OR AB fishes OR SU fishes))) OR (TI hogfish* OR AB hogfish* OR SU hogfish*) OR (TI "hog fish*" OR AB "hog fish*" OR SU "hog fish*") OR (TI houndfish* OR AB houndfish* OR SU houndfish*) OR (TI "hound fish*" OR AB "hound fish*" OR SU "hound fish*") OR (TI hualca* OR AB hualca* OR SU hualca*) OR (TI icefish* OR AB icefish* OR SU icefish*) OR (TI "ice fish*" OR AB "ice fish*" OR SU "ice fish*") OR (TI inaha* OR AB inaha* OR SU inaha*) OR (TI isopod* OR AB isopod* OR SU isopod*) OR (TI jellyfish* OR AB jellyfish* OR SU jellyfish*) OR (TI "jelly fish*" OR AB "jelly fish*" OR SU "jelly fish*") OR (TI jewfish* OR AB jewfish* OR SU jewfish*) OR (TI "jew fish*" OR AB "jew fish*" OR SU "jew fish*") OR (TI jobfish* OR AB jobfish* OR SU jobfish*) OR (TI "job fish*" OR AB "job fish*" OR SU "job fish*") OR (TI kahawai* OR AB kahawai* OR SU kahawai*) OR (TI kajuli* OR AB kajuli* OR SU kajuli*) OR (TI kanpachi* OR AB kanpachi* OR SU kanpachi*) OR (TI kelpfish* OR AB kelpfish* OR SU kelpfish*) OR (TI "kelp fish*" OR AB "kelp fish*" OR SU "kelp fish*") OR (TI killifish* OR AB killifish* OR SU killifish*) OR (TI "killi fish*" OR AB "killi fish*" OR SU "killi fish*") OR (TI kingfish* OR AB kingfish* OR SU kingfish*) OR (TI "king fish*" OR AB "king fish*" OR SU "king fish*") OR (TI kingklip* OR AB kingklip* OR SU kingklip*) OR (TI "king klip*" OR AB "king klip*" OR SU "king klip*") OR (TI knifefish* OR AB knifefish* OR SU knifefish*) OR (TI "knife fish*" OR AB "knife fish*" OR SU "knife fish*") OR (TI knifejaw* OR AB knifejaw* OR SU knifejaw*) OR (TI "knife jaw*" OR AB "knife jaw*" OR SU "knife jaw*") OR (TI krill# OR AB krill# OR SU krill#) OR (TI ladyfish* OR AB ladyfish* OR SU ladyfish*) OR (TI "lady fish*" OR AB "lady fish*" OR SU "lady fish*") OR (TI lancet* OR AB lancet* OR SU lancet*) OR (TI langostino* OR AB langostino* OR SU langostino*) OR (TI leaffish* OR AB leaffish* OR SU leaffish*) OR (TI "leaf fish*" OR AB "leaf fish*" OR SU "leaf fish*") OR (TI leatherjacket* OR AB leatherjacket* OR SU leatherjacket*) OR (TI "leather jacket*" OR AB

		"leather jacket*" OR SU "leather jacket*") OR (TI limpet* OR AB limpet* OR SU limpet*) OR ((TI ling# OR AB ling# OR SU ling#) AND ((TI fish OR AB fish OR SU fish) OR (TI fishes OR AB fishes OR SU fishes))) OR (TI lingcod* OR AB lingcod* OR SU lingcod*) OR (TI lionfish* OR AB lionfish* OR SU lionfish*) OR (TI "lion fish*" OR AB "lion fish*" OR SU "lion fish*") OR (TI lizardfish* OR AB lizardfish* OR SU lizardfish*) OR (TI "lizard fish*" OR AB "lizard fish*" OR SU "lizard fish*") OR (TI loach* OR AB loach* OR SU loach*) OR (TI lobster* OR AB lobster* OR SU lobster*) OR (TI louvar* OR AB louvar* OR SU louvar*) OR (TI lumpfish* OR AB lumpfish* OR SU lumpfish*) OR (TI "lump fish*" OR AB "lump fish*" OR SU "lump fish*") OR (TI lyretail* OR AB lyretail* OR SU lyretail*) OR (TI "lyre tail*" OR AB "lyre tail*" OR SU "lyre tail*") OR (TI mackerel* OR AB mackerel* OR SU mackerel*) OR (TI manta# OR AB manta# OR SU manta#) OR (TI margate* OR AB margate* OR SU margate*) OR (TI marlin# OR AB marlin# OR SU marlin#) OR (TI megrim* OR AB megrim* OR SU megrim*) OR (TI menhaden* OR AB menhaden* OR SU menhaden*) OR (TI merex* OR AB merex* OR SU merex*) OR (TI miiuy* OR AB miiuy* OR SU miiuy*) OR (TI milkfish* OR AB milkfish* OR SU milkfish*) OR (TI "milk fish*" OR AB "milk fish*" OR SU "milk fish*") OR (TI minnow* OR AB minnow* OR SU minnow*) OR (TI moga# OR AB moga# OR SU moga#) OR (TI mojarra* OR AB mojarra* OR SU mojarra*) OR (TI moki# OR AB moki# OR SU moki#) OR (TI mola OR AB mola OR SU mola) OR (TI molas OR AB molas OR SU molas) OR (TI monkfish* OR AB monkfish* OR SU monkfish*) OR (TI "monk fish*" OR AB "monk fish*" OR SU "monk fish*") OR (TI mooneye* OR AB mooneye* OR SU mooneye*) OR (TI "moon eye*" OR AB "moon eye*" OR SU "moon eye*") OR (TI moonfish* OR AB moonfish* OR SU moonfish*) OR (TI "moon fish*" OR AB "moon fish*" OR SU "moon fish*") OR (TI moonsnail* OR AB moonsnail* OR SU moonsnail*) OR (TI "moon snail*" OR AB "moon snail*" OR SU "moon snail*") OR (TI morwong* OR AB morwong* OR SU morwong*) OR (TI mrigal* OR AB mrigal* OR SU mrigal*) OR (TI mullet# OR AB mullet# OR SU mullet#) OR (TI murex OR AB murex OR SU murex) OR (TI murexes OR AB murexes OR SU murexes) OR (TI muskellunge* OR AB muskellunge* OR SU muskellunge*) OR (TI mussel# OR AB mussel# OR SU mussel#) OR (TI mystus OR AB mystus OR SU mystus) OR (TI needlefish* OR AB needlefish* OR SU needlefish*) OR (TI "needle fish*" OR AB "needle fish*" OR SU "needle fish*") OR (TI nodoguro* OR AB nodoguro* OR SU nodoguro*) OR (TI noodlefish* OR AB noodlefish* OR SU noodlefish*) OR (TI "noodle fish*" OR AB "noodle fish*" OR SU "noodle fish*") OR (TI octopus* OR AB octopus* OR SU octopus*) OR (TI octopi OR AB octopi OR SU octopi) OR (TI oilfish* OR AB oilfish* OR SU oilfish*) OR (TI "oil fish*" OR AB "oil fish*" OR SU "oil fish*") OR (TI opah# OR AB opah# OR SU opah#) OR (TI opaleye* OR AB opaleye* OR SU opaleye*) OR (TI "opal eye*" OR AB "opal eye*" OR SU "opal eye*") OR (TI oscar# OR AB oscar# OR SU oscar#) OR (TI oyster# OR AB oyster# OR SU oyster#) OR (TI pabdah* OR AB pabdah* OR SU pabdah*) OR (TI packoo* OR AB packoo* OR SU packoo*) OR (TI pacu# OR AB pacu# OR SU pacu#) OR (TI paddlefish* OR AB paddlefish* OR SU paddlefish*) OR (TI "paddle fish*" OR AB "paddle fish*" OR SU "paddle fish*") OR (TI paima# OR AB paima# OR SU paima#) OR (TI pangasius* OR AB pangasius* OR SU pangasius*) OR (TI pargo# OR AB pargo# OR SU pargo#) OR (TI parrotfish* OR AB parrotfish* OR SU parrotfish*) OR (TI "parrot fish*" OR AB "parrot fish*" OR SU "parrot fish*") OR (TI pearlspot* OR AB pearlspot* OR SU pearlspot*) OR (TI "pearl spot*" OR AB "pearl spot*" OR SU "pearl spot*") OR (TI penshell* OR AB penshell* OR SU penshell*) OR (TI "pen shell*" OR AB "pen shell*" OR SU "pen shell*") OR (TI perch OR AB perch OR SU perch) OR (TI perches OR AB perches OR SU perches) OR (TI periwinkle# OR AB periwinkle# OR SU periwinkle#) OR (TI picarel* OR AB picarel* OR SU picarel*) OR (TI pickerel* OR AB pickerel* OR SU

		pickerel*) OR (TI pike# OR AB pike# OR SU pike#) OR (TI pikeperch* OR AB pikeperch* OR SU pikeperch*) OR (TI pilchard* OR AB pilchard* OR SU pilchard*) OR (TI pintado* OR AB pintado* OR SU pintado*) OR (TI pipefish* OR AB pipefish* OR SU pipefish*) OR (TI "pipe fish*" OR AB "pipe fish*" OR SU "pipe fish*") OR (TI piramutaba* OR AB piramutaba* OR SU piramutaba*) OR (TI pirarucu* OR AB pirarucu* OR SU pirarucu*) OR (TI plaice* OR AB plaice* OR SU plaice*) OR (TI pleco# OR AB pleco# OR SU pleco#) OR (TI pollock# OR AB pollock# OR SU pollock#) OR (TI pomfret# OR AB pomfret# OR SU pomfret#) OR (TI pompanito* OR AB pompanito* OR SU pompanito*) OR (TI pompano* OR AB pompano* OR SU pompano*) OR (TI ponyfish* OR AB ponyfish* OR SU ponyfish*) OR (TI "pony fish*" OR AB "pony fish*" OR SU "pony fish*") OR (TI porgy# OR AB porgy# OR SU porgy#) OR (TI porgies OR AB porgies OR SU porgies) OR (TI porkfish* OR AB porkfish* OR SU porkfish*) OR (TI "pork fish*" OR AB "pork fish*" OR SU "pork fish*") OR (TI potasi# OR AB potasi# OR SU potasi#) OR (TI pout# OR AB pout# OR SU pout#) OR (TI prawn# OR AB prawn# OR SU prawn#) OR (TI puffer* OR AB puffer* OR SU puffer*) OR (TI punti# OR AB punti# OR SU punti#) OR (TI quahog* OR AB quahog* OR SU quahog*) OR (TI queenfish* OR AB queenfish* OR SU queenfish*) OR (TI "queen fish*" OR AB "queen fish*" OR SU "queen fish*") OR ((TI racehorse# OR AB racehorse# OR SU racehorse#) AND ((TI fish OR AB fish OR SU fish) OR (TI fishes OR AB fishes OR SU fishes))) OR (((TI ray OR AB ray OR SU ray) OR (TI rays OR AB rays OR SU rays)) AND ((TI fish OR AB fish OR SU fish) OR (TI fishes OR AB fishes OR SU fishes))) OR (TI redfish* OR AB redfish* OR SU redfish*) OR (TI "red fish*" OR AB "red fish*" OR SU "red fish*") OR (TI redhorse* OR AB redhorse* OR SU redhorse*) OR (TI "red horse*" OR AB "red horse*" OR SU "red horse*") OR (TI remora# OR AB remora# OR SU remora#) OR (TI rita# OR AB rita# OR SU rita#) OR (TI rockfish* OR AB rockfish* OR SU rockfish*) OR (TI "rock fish*" OR AB "rock fish*" OR SU "rock fish*") OR (TI rockling* OR AB rockling* OR SU rockling*) OR (TI rohu# OR AB rohu# OR SU rohu#) OR (TI roosterfish* OR AB roosterfish* OR SU roosterfish*) OR (TI "rooster fish*" OR AB "rooster fish*" OR SU "rooster fish*") OR (TI rosefish* OR AB rosefish* OR SU rosefish*) OR (TI "rose fish*" OR AB "rose fish*" OR SU "rose fish*") OR (TI roughback* OR AB roughback* OR SU roughback*) OR (TI "rough back*" OR AB "rough back*" OR SU "rough back*") OR (TI roughies OR AB roughies OR SU roughies) OR (TI roughy* OR AB roughy* OR SU roughy*) OR (TI ruff OR AB ruff OR SU ruff) OR ((TI runner# OR AB runner# OR SU runner#) AND ((TI fish OR AB fish OR SU fish) OR (TI fishes OR AB fishes OR SU fishes))) OR (TI sablefish* OR AB sablefish* OR SU sablefish*) OR (TI "sable fish*" OR AB "sable fish*" OR SU "sable fish*") OR (TI sailfish* OR AB sailfish* OR SU sailfish*) OR (TI "sail fish*" OR AB "sail fish*" OR SU "sail fish*") OR (TI salmon* OR AB salmon* OR SU salmon*) OR (TI sampa# OR AB sampa# OR SU sampa#) OR (TI sanddab* OR AB sanddab* OR SU sanddab*) OR (TI sandeel* OR AB sandeel* OR SU sandeel*) OR ((TI sander# OR AB sander# OR SU sander#) AND ((TI fish OR AB fish OR SU fish) OR (TI fishes OR AB fishes OR SU fishes))) OR (TI sandfish* OR AB sandfish* OR SU sandfish*) OR (TI "sand fish*" OR AB "sand fish*" OR SU "sand fish*") OR (TI sandperch* OR AB sandperch* OR SU sandperch*) OR (TI sardine# OR AB sardine# OR SU sardine#) OR (TI sargo# OR AB sargo# OR SU sargo#) OR (TI sauger# OR AB sauger# OR SU sauger#) OR (TI sauries OR AB sauries OR SU sauries) OR (TI saury* OR AB saury* OR SU saury*) OR (TI sawfish* OR AB sawfish* OR SU sawfish*) OR (TI "saw fish*" OR AB "saw fish*" OR SU "saw fish*") OR (TI scad# OR AB scad# OR SU scad#) OR (TI scallop# OR AB scallop# OR SU scallop#) OR (TI scamp# OR AB scamp# OR SU scamp#) OR (TI schoolmaster* OR AB schoolmaster* OR SU schoolmaster*) OR (TI scorpionfish* OR AB scorpionfish* OR SU

		scorpionfish*) OR (TI "scorpion fish*" OR AB "scorpion fish*" OR SU "scorpion fish*") OR (TI scup OR AB scup OR SU scup) OR (TI scups OR AB scups OR SU scups) OR (TI scupper# OR AB scupper# OR SU scupper#) OR (TI seabass* OR AB seabass* OR SU seabass*) OR (TI seabob* OR AB seabob* OR SU seabob*) OR (TI "sea bob*" OR AB "sea bob*" OR SU "sea bob*") OR (TI seabream* OR AB seabream* OR SU seabream*) OR (TI searobin* OR AB searobin* OR SU searobin*) OR (TI "sea robin*" OR AB "sea robin*" OR SU "sea robin*") OR (TI seasnail* OR AB seasnail* OR SU seasnail*) OR (TI "sea snail*" OR AB "sea snail*" OR SU "sea snail*") OR (TI shad OR AB shad OR SU shad) OR (TI shads OR AB shads OR SU shads) OR (TI shadefish* OR AB shadefish* OR SU shadefish*) OR (TI "shade fish*" OR AB "shade fish*" OR SU "shade fish*") OR (TI shark# OR AB shark# OR SU shark#) OR (TI sharksucker# OR AB sharksucker# OR SU sharksucker#) OR (TI sheatfish* OR AB sheatfish* OR SU sheatfish*) OR (TI "sheat fish*" OR AB "sheat fish*" OR SU "sheat fish*") OR (TI sheep#head# OR AB sheep#head# OR SU sheep#head#) OR (TI "sheep# head#" OR AB "sheep# head#" OR SU "sheep# head#") OR (TI shellfish* OR AB shellfish* OR SU shellfish*) OR (TI "shell fish*" OR AB "shell fish*" OR SU "shell fish*") OR (TI shiner# OR AB shiner# OR SU shiner#) OR (TI shrimp# OR AB shrimp# OR SU shrimp#) OR (TI sild OR AB sild OR SU sild) OR (TI silds OR AB silds OR SU silds) OR (TI sillago* OR AB sillago* OR SU sillago*) OR (TI silverside* OR AB silverside* OR SU silverside*) OR (TI "silver side*" OR AB "silver side*" OR SU "silver side*") OR (TI skate# OR AB skate# OR SU skate#) OR (TI skilfish* OR AB skilfish* OR SU skilfish*) OR (TI "skil fish*" OR AB "skil fish*" OR SU "skil fish*") OR (TI slipmouth* OR AB slipmouth* OR SU slipmouth*) OR (TI "slip mouth*" OR AB "slip mouth*" OR SU "slip mouth*") OR (TI slipperysnail* OR AB slipperysnail* OR SU slipperysnail*) OR (TI "slippery snail*" OR AB "slippery snail*" OR SU "slippery snail*") OR ((TI smelt* OR AB smelt* OR SU smelt*) AND ((TI fish OR AB fish OR SU fish) OR (TI fishes OR AB fishes OR SU fishes))) OR (TI smoothhound* OR AB smoothhound* OR SU smoothhound*) OR (TI "smooth hound*" OR AB "smooth hound*" OR SU "smooth hound*") OR (TI snail# OR AB snail# OR SU snail#) OR (TI snakehead# OR AB snakehead# OR SU snakehead#) OR (TI snapper# OR AB snapper# OR SU snapper#) OR (TI snook# OR AB snook# OR SU snook#) OR (TI soldierfish* OR AB soldierfish* OR SU soldierfish*) OR (TI "soldier fish*" OR AB "soldier fish*" OR SU "soldier fish*") OR ((TI sole* OR AB sole* OR SU sole*) AND ((TI fish OR AB fish OR SU fish) OR (TI fishes OR AB fishes OR SU fishes))) OR (TI s#rubi# OR AB s#rubi# OR SU s#rubi#) OR (TI spadefish* OR AB spadefish* OR SU spadefish*) OR (TI "spade fish*" OR AB "spade fish*" OR SU "spade fish*") OR (TI spearfish* OR AB spearfish* OR SU spearfish*) OR (TI "spear fish*" OR AB "spear fish*" OR SU "spear fish*") OR (TI spinef??t* OR AB spinef??t* OR SU spinef??t*) OR (TI "spine f??t*" OR AB "spine f??t*" OR SU "spine f??t*") OR ((TI spot* OR AB spot* OR SU spot*) AND ((TI fish OR AB fish OR SU fish) OR (TI fishes OR AB fishes OR SU fishes))) OR (TI sprat OR AB sprat OR SU sprat) OR (TI sprats OR AB sprats OR SU sprats) OR (TI squid# OR AB squid# OR SU squid#) OR (TI squirrelfish* OR AB squirrelfish* OR SU squirrelfish*) OR (TI "squirrel fish*" OR AB "squirrel fish*" OR SU "squirrel fish*") OR (TI stargazer* OR AB stargazer* OR SU stargazer*) OR (TI "star gazer*" OR AB "star gazer*" OR SU "star gazer*") OR ((TI stinging OR AB stinging OR SU stinging) AND ((TI fish OR AB fish OR SU fish) OR (TI fishes OR AB fishes OR SU fishes))) OR (TI stingray* OR AB stingray* OR SU stingray*) OR (TI "sting ray*" OR AB "sting ray*" OR SU "sting ray*") OR (TI straptail* OR AB straptail* OR SU straptail*) OR (TI "strap tail*" OR AB "strap tail*" OR SU "strap tail*") OR (TI sturgeon* OR AB sturgeon* OR SU sturgeon*) OR ((TI sucker# OR AB sucker# OR SU sucker#) AND ((TI fish OR AB fish OR SU fish) OR (TI fishes OR AB fishes OR SU fishes))) OR (TI suckerfish*

		OR AB suckerfish* OR SU suckerfish*) OR (TI sunfish* OR AB sunfish* OR SU sunfish*) OR (TI "sun fish*" OR AB "sun fish*" OR SU "sun fish*") OR (TI surfclam* OR AB surfclam* OR SU surfclam*) OR (TI "surf clam*" OR AB "surf clam*" OR SU "surf clam*") OR (TI surfperch* OR AB surfperch* OR SU surfperch*) OR (TI surgeonfish* OR AB surgeonfish* OR SU surgeonfish*) OR (TI "surgeon fish*" OR AB "surgeon fish*" OR SU "surgeon fish*") OR (TI sutchi# OR AB sutchi# OR SU sutchi#) OR (TI swai# OR AB swai# OR SU swai#) OR (TI sweetlip* OR AB sweetlip* OR SU sweetlip*) OR (TI "sweet lip*" OR AB "sweet lip*" OR SU "sweet lip*") OR (TI swordfish* OR AB swordfish* OR SU swordfish*) OR (TI "sword fish*" OR AB "sword fish*" OR SU "sword fish*") OR (TI tambaqui* OR AB tambaqui* OR SU tambaqui*) OR (TI tang OR AB tang OR SU tang) OR (TI tangs OR AB tangs OR SU tangs) OR (TI tarakihi* OR AB tarakihi* OR SU tarakihi*) OR (TI tarpon# OR AB tarpon# OR SU tarpon#) OR (TI tautog* OR AB tautog* OR SU tautog*) OR (TI tench OR AB tench OR SU tench) OR (TI tenches OR AB tenches OR SU tenches) OR (TI therapon# OR AB therapon# OR SU therapon#) OR (TI thornyhead* OR AB thornyhead* OR SU thornyhead*) OR (TI "thorny head*" OR AB "thorny head*" OR SU "thorny head*") OR (TI threadfin* OR AB threadfin* OR SU threadfin*) OR (TI "thread fin*" OR AB "thread fin*" OR SU "thread fin*") OR (TI tigerfish* OR AB tigerfish* OR SU tigerfish*) OR (TI "tiger fish*" OR AB "tiger fish*" OR SU "tiger fish*") OR (TI tigerperch* OR AB tigerperch* OR SU tigerperch*) OR (TI tilapia* OR AB tilapia* OR SU tilapia*) OR (TI tilefish* OR AB tilefish* OR SU tilefish*) OR (TI "tile fish*" OR AB "tile fish*" OR SU "tile fish*") OR ((TI tinfoil* OR AB tinfoil* OR SU tinfoil*) AND ((TI fish OR AB fish OR SU fish) OR (TI fishes OR AB fishes OR SU fishes))) OR (TI toadfish* OR AB toadfish* OR SU toadfish*) OR (TI "toad fish*" OR AB "toad fish*" OR SU "toad fish*") OR (TI tomcod* OR AB tomcod* OR SU tomcod*) OR (TI tomtate* OR AB tomtate* OR SU tomtate*) OR (TI to#ng#esole* OR AB to#ng#esole* OR SU to#ng#esole*) OR (TI "to#ng#e sole*" OR AB "to#ng#e sole*" OR SU "to#ng#e sole*") OR (TI toothfish* OR AB toothfish* OR SU toothfish*) OR (TI "tooth fish*" OR AB "tooth fish*" OR SU "tooth fish*") OR ((TI torpedo* OR AB torpedo* OR SU torpedo*) AND ((TI fish OR AB fish OR SU fish) OR (TI fishes OR AB fishes OR SU fishes))) OR (TI tra OR AB tra OR SU tra) OR (TI tras OR AB tras OR SU tras) OR (TI trevallies OR AB trevallies OR SU trevallies) OR (TI trevally* OR AB trevally* OR SU trevally*) OR (TI triggerfish* OR AB triggerfish* OR SU triggerfish*) OR (TI "trigger fish*" OR AB "trigger fish*" OR SU "trigger fish*") OR (TI tripletail* OR AB tripletail* OR SU tripletail*) OR (TI "tripe tail*" OR AB "tripe tail*" OR SU "tripe tail*") OR (TI trout# OR AB trout# OR SU trout#) OR (TI trumpeter* OR AB trumpeter* OR SU trumpeter*) OR (TI trumpetfish* OR AB trumpetfish* OR SU trumpetfish*) OR (TI "trumpet fish*" OR AB "trumpet fish*" OR SU "trumpet fish*") OR (TI trunkfish* OR AB trunkfish* OR SU trunkfish*) OR (TI "trunk fish*" OR AB "trunk fish*" OR SU "trunk fish*") OR (TI tullibee* OR AB tullibee* OR SU tullibee*) OR (TI tuna OR AB tuna OR SU tuna) OR (TI tunas OR AB tunas OR SU tunas) OR (TI tunicata* OR AB tunicata* OR SU tunicata*) OR (TI turbot# OR AB turbot# OR SU turbot#) OR (TI turtle# OR AB turtle# OR SU turtle#) OR (TI unicornfish* OR AB unicornfish* OR SU unicornfish*) OR (TI "unicorn fish*" OR AB "unicorn fish*" OR SU "unicorn fish*") OR (TI urchin# OR AB urchin# OR SU urchin#) OR (TI vendace* OR AB vendace* OR SU vendace*) OR (TI volute# OR AB volute# OR SU volute#) OR (TI wahoo* OR AB wahoo* OR SU wahoo*) OR (TI walleye* OR AB walleye* OR SU walleye*) OR (TI "wall eye*" OR AB "wall eye*" OR SU "wall eye*") OR (TI warehou OR AB warehou OR SU warehou) OR (TI weakfish* OR AB weakfish* OR SU weakfish*) OR (TI "weak fish*" OR AB "weak fish*" OR SU "weak fish*") OR (TI weever* OR AB weever* OR SU weever*) OR (TI whale# OR AB whale# OR SU whale#) OR (TI whalemeat# OR AB whalemeat# OR SU whalemeat#) OR (TI whelk# OR AB whelk# OR SU

		whelk#) OR (TI whiff# OR AB whiff# OR SU whiff#) OR (TI whiskerfish* OR AB whiskerfish* OR SU whiskerfish*) OR (TI "whisker fish*" OR AB "whisker fish*" OR SU "whisker fish*") OR (TI whitefish* OR AB whitefish* OR SU whitefish*) OR (TI "white fish*" OR AB "white fish*" OR SU "white fish*") OR (TI whiting* OR AB whiting* OR SU whiting*) OR (TI wolffish* OR AB wolffish* OR SU wolffish*) OR (TI "wolf fish*" OR AB "wolf fish*" OR SU "wolf fish*") OR (TI wrasse# OR AB wrasse# OR SU wrasse#) OR (TI yellowtail* OR AB yellowtail* OR SU yellowtail*) OR (TI "yellow tail*" OR AB "yellow tail*" OR SU "yellow tail*") OR (TI zander# OR AB zander# OR SU zander#) OR (TI zungaro* OR AB zungaro* OR SU zungaro*) OR (TI "lau lau*" OR AB "lau lau*" OR SU "lau lau*") OR (TI "mahi mahi*" OR AB "mahi mahi*" OR SU "mahi mahi*") OR (TI "sergeant major*" OR AB "sergeant major*" OR SU "sergeant major*") OR (TI "sea cucumber*" OR AB "sea cucumber*" OR SU "sea cucumber*") OR (TI "american smelt*" OR AB "american smelt*" OR SU "american smelt*") O
pregnancy	S2	(DE "Pregnancy" OR DE "Adolescent Pregnancy" OR DE "Pregnancy Outcomes" OR DE "Primipara" OR DE "Induced Abortion" OR DE "Spontaneous Abortion" OR DE "Birth" OR DE "Birth Weight" OR DE "Caesarean Birth" OR DE "Labor (Childbirth)" OR DE "Natural Childbirth" OR DE "Premature Birth" OR DE "Antepartum Period" OR DE "Peripartum Period" OR DE "Intrapartum Period" OR DE "Postnatal Period" OR DE "Obstetrical Complications" OR DE "Gestational Diabetes" OR DE "Preeclampsia" OR DE "Fetus" OR DE "Prenatal Exposure" OR DE "Prenatal Development" OR DE "Perinatal Period") OR ((TI pregnan* OR AB pregnan* OR SU pregnan*) OR (TI prenatal* OR AB prenatal* OR SU prenatal*) OR (TI "post natal*" OR AB "post natal*" OR SU "post natal*") OR (TI postnatal* OR AB postnatal* OR SU postnatal*) OR (TI "post natal*" OR AB "post natal*" OR SU "post natal*") OR (TI antenatal* OR AB antenatal* OR SU antenatal*) OR (TI "ante natal*" OR AB "ante natal*" OR SU "ante natal*") OR (TI perinatal* OR AB perinatal* OR SU perinatal*) OR (TI "peri natal*" OR AB "peri natal*" OR SU "peri natal*") OR (TI postpartum OR AB postpartum OR SU postpartum) OR (TI "post partum" OR AB "post partum" OR SU "post partum") OR (TI f#etus* OR AB f#etus* OR SU f#etus*) OR (TI f#etal OR AB f#etal OR SU f#etal) OR (TI maternal OR AB maternal OR SU maternal) OR (TI gravid* OR AB gravid* OR SU gravid*) OR (TI birth* OR AB birth* OR SU birth*))
lactation	S3	(DE "Lactation" OR DE "Breast Feeding" OR DE "Weaning") OR ((TI lactat* OR AB lactat* OR SU lactat*) OR (TI breastfe* OR AB breastfe* OR SU breastfe*) OR ((TI breast OR AB breast OR SU breast) W1 ((TI fed OR AB fed OR SU fed) OR (TI feed* OR AB feed* OR SU feed*))) OR (((TI human# OR AB human# OR SU human#) OR (TI breast* OR AB breast* OR SU breast*) OR (TI maternal OR AB maternal OR SU maternal) OR (TI mother* OR AB mother* OR SU mother*)) N2 (TI milk* OR AB milk* OR SU milk*)) OR (TI breastmilk# OR AB breastmilk# OR SU breastmilk#) OR (TI colostrum OR AB colostrum OR SU colostrum))
infants	S4	(DE "Infant Development" OR DE "Infant Temperament" OR DE "Neonatal Development" OR DE "Neonatal Period" OR DE "Neonatal Disorders") OR ((((TI 0 OR AB 0 OR SU 0) OR (TI 1 OR AB 1 OR SU 1) OR (TI 2 OR AB 2 OR SU 2) OR (TI zero OR AB zero OR SU zero) OR (TI one OR AB one OR SU one) OR (TI two OR AB two OR SU two)) N5 ((TI age# OR AB age# OR SU age#) OR (TI year# OR AB year# OR SU year#) OR (TI old OR AB old OR SU old))) OR (((TI 0 OR AB 0 OR SU 0) OR (TI 1 OR AB 1 OR SU 1) OR (TI 2 OR AB 2 OR SU 2) OR (TI zero OR AB zero OR SU zero) OR (TI one OR AB one OR SU one) OR (TI two OR AB two OR SU two)) N5 ((TI yr OR AB yr OR SU yr) OR (TI yrs OR AB yrs OR SU yrs) OR (TI y OR AB y OR SU y) OR (TI ys OR AB ys OR SU ys))) OR (((TI month# OR AB month# OR SU month#) OR (TI mnth# OR AB mnth# OR SU mnth#) OR (TI mth# OR AB mth# OR SU mth#) OR (TI ms OR AB ms OR SU ms) OR (TI m OR AB m OR SU m) OR (TI

		week# OR AB week# OR SU week#) OR (TI wks OR AB wks OR SU wks) OR (TI wk OR AB wk OR SU wk) OR W1 OR (TI ws OR AB ws OR SU ws) OR (TI day# OR AB day# OR SU day#) OR (TI ds OR AB ds OR SU ds) OR (TI d OR AB d OR SU d)) N5 ((TI old OR AB old OR SU old) OR (TI age# OR AB age# OR SU age#))) OR (TI infan* OR AB infan* OR SU infan*) OR (TI baby OR AB baby OR SU baby) OR (TI babies OR AB babies OR SU babies) OR (TI newborn* OR AB newborn* OR SU newborn*) OR (TI "new born*" OR AB "new born*" OR SU "new born*") OR (TI neonat* OR AB neonat* OR SU neonat*) OR (TI "neo nat*" OR AB "neo nat*" OR SU "neo nat*"))
	S5	S2 OR S3 OR S4
lead	S6	(DE "Lead (Metal)" OR DE "Lead Poisoning" OR SU lead OR TM lead) OR ((TI pb OR AB pb OR SU pb) OR (TI 208Pb OR AB 208Pb OR SU 208Pb) OR (TI organolead* OR AB organolead* OR SU organolead*) OR (TI plumbum OR AB plumbum OR SU plumbum) OR (TI plumbic* OR AB plumbic* OR SU plumbic*) OR (TI plumbate* OR AB plumbate* OR SU plumbate*) OR (TI plumbous OR AB plumbous OR SU plumbous) OR (TI tetraethyllead OR AB tetraethyllead OR SU tetraethyllead) OR ((TI lead OR AB lead OR SU lead) N8 ((TI 208 OR AB 208 OR SU 208) OR (TI metal* OR AB metal* OR SU metal*) OR (TI organometal* OR AB organometal* OR SU organometal*) OR (TI cation* OR AB cation* OR SU cation*) OR (TI ion* OR AB ion* OR SU ion*) OR (TI isotop* OR AB isotop* OR SU isotop*) OR (TI radioisotop* OR AB radioisotop* OR SU radioisotop*) OR (TI element* OR AB element* OR SU element*) OR (TI microelement* OR AB microelement* OR SU microelement*) OR (TI organic OR AB organic OR SU organic) OR (TI inorganic OR AB inorganic OR SU inorganic) OR (TI ore OR AB ore OR SU ore) OR (TI blood* OR AB blood* OR SU blood*) OR (TI chemical# OR AB chemical# OR SU chemical#) OR (TI poison* OR AB poison* OR SU poison*) OR (TI contam* OR AB contam* OR SU contam*))))
search structure	S7	S1 AND S5 AND S6

Supplementary Table 3: Inclusion and exclusion criteria for a systematic review of the association between perinatal exposure to lead from seafood consumption and child neurodevelopment

Category	Inclusion Criteria	Exclusion Criteria
**Population:**<br>• **Country**<br>• **Health status**<br>• **Exp pop**<br>• **Outcome pop**<br>• **Species**	● Country: Individuals living in countries ranked as high or very high on the human development index during the study.<br>● Exposed population: Individuals in the general population who are pregnant or lactating, infants, children, or adolescents up to age 18 years.<br>● Outcome population: Children and adolescents (up to age 18 years).<br>● Species: Humans only	● Health status: Studies exclusively of participants with a chronic condition, hospitalized with an illness or injury. Examples include:<br>○ Diabetes (not including gestational diabetes)<br>○ Cancer<br>○ Cardiometabolic disorders<br>○ Chronic kidney disease<br>○ Malabsorption (any disorder that causes malabsorption from the gastrointestinal tract)<br>○ Asthma<br>● Species: Non-human
**Exposure:**<br>**Seafood intake + Toxicant**	Must contain Exposure 1 AND Exposure 2<br>**Exposure 1: Seafood consumption:**<br>● Types (e.g., salmon, tuna, bass)<br>● Sources (e.g., sea, fresh water, farmed, canned, wild)<br>● Amount (e.g., ounces per day, grams per meal)<br>● Frequency (e.g., daily, twice a week)<br>● Duration (e.g., length of time-consuming seafood)<br>● Preparation (e.g., fried, baked)<br>● Timing (e.g., by trimester, age)<br>**Exposure 2: Toxin or toxicants**<br>○ Lead (Pb)	● Studies that do not report on toxicant exposure in fish AND seafood consumption<br>● Supplements<br>● Infant formula<br>● Cyanobacteria<br>● Ciguatera<br>● Scombroid<br>● Domoic acid (red algae)
**Comparator**	● Exposure to different levels of the toxins or toxicants of interest; No exposure to the toxins or toxicants of interest<br>● Different types, sources, amounts, frequencies, durations, preparations, or timings of seafood consumption; No seafood consumption	● No comparator

**Outcomes**	Neurodevelopment related:<br>● Cognitive development<br>● Motor development and movement<br>● Behavioral issues<br>● Attention<br>● Attention deficit hyperactivity disorder<br>● Autism	
**Study designs**	• Randomized controlled trials<br>• Controlled (nonrandomized) trials<br>• Cohort (observational) studies, prospective or retrospective<br>• Case-cohort studies (Nested case-control)<br>• Case-control studies<br>• Before-after studies	● Studies without primary data, such as systematic reviews, narrative reviews, editorials, and commentaries<br>● Case reports<br>● Studies reported in theses or conference abstracts only.<br>● Studies not reported in English

Supplementary Figure 1. Eligible articles for the systematic review based on the investigated relationships between exposures and outcomes

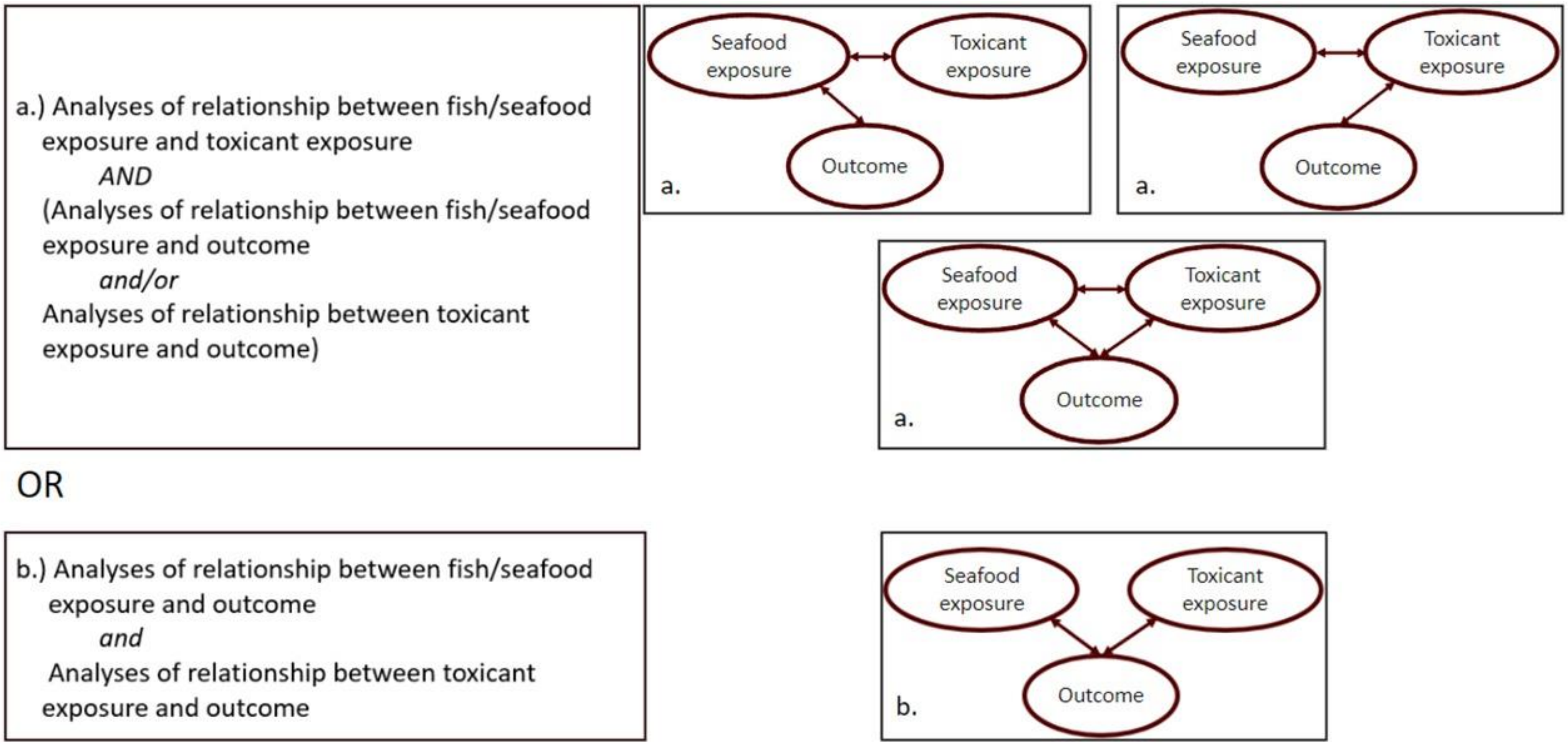

Supplementary Figure 2. Mechanism of co-pollutant and other nutrients confounding

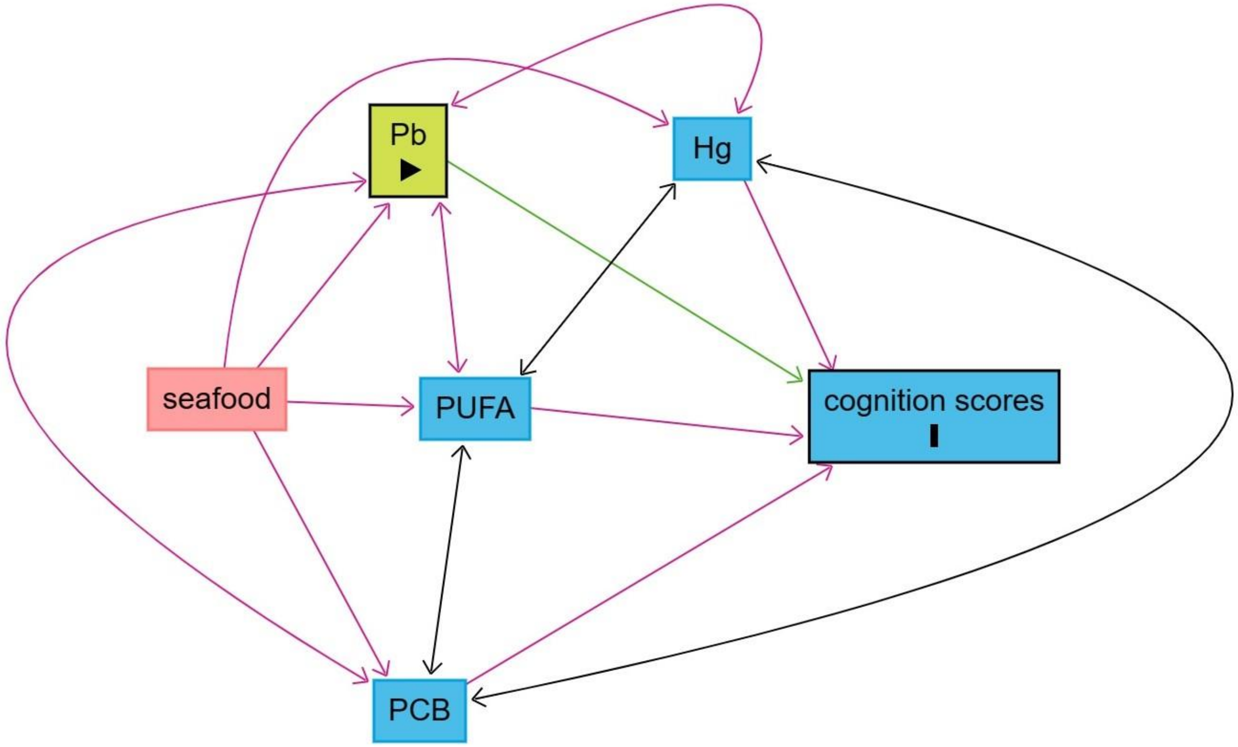

Main exposure is Pb in the green box with a triangle; outcome is cognition scores in the blue box with rectangle; and, seafood intake, Mercury, PUFAs and PCBs serve as potential confounders. Adjusting for Mercury (Hg), PUFAs and PCBs (and other co-pollutants) would close the backdoor pathways from Pb to neurodevelopmental outcome.
Reference: Johannes Textor, Benito van der Zander, Mark K. Gilthorpe, Maciej Liskiewicz, George T.H. Ellison.
Robust causal inference using directed acyclic graphs: the R package 'dagitty'.
International Journal of Epidemiology 45(6):1887-1894, 2016.
